# Lewis Acid-Base Adducts of α-Amino Acid-Derived Silaheterocycles and *N*-Methylimidazole

**DOI:** 10.3390/molecules28237816

**Published:** 2023-11-28

**Authors:** Anne Seidel, Robert Gericke, Beate Kutzner, Jörg Wagler

**Affiliations:** 1 Institut für Anorganische Chemie, TU Bergakademie Freiberg, D-09596 Freiberg, Germany; anne.seidel@chemie.tu-freiberg.de (A.S.); beate.kutzner@chemie.tu-freiberg.de (B.K.); 2 Institute of Resource Ecology, Helmholtz-Zentrum Dresden-Rossendorf eV, D-01328 Dresden, Germany

**Keywords:** bidentate ligands, deuterium transfer, hypercoordination, quantum chemical calculations, silicon, X-ray diffraction

## Abstract

In chloroform solution, the reaction of bis(tert-butylamino)dimethylsilane ((*t*BuNH)_2_SiMe_2_) and an α-amino acid (α-amino isobutyric acid, **H_2_Aib**; D-phenylglycine, **H_2_Phg**; L-valine, **H_2_Val**) in the presence of *N*-methylimidazole (NMI) gave rise to the formation of the pentacoordinate silicon complexes **(Aib)SiMe_2_-NMI**, **(Phg)SiMe_2_-NMI** and **(Val)SiMe_2_-NMI**, respectively. Therein, the amino acid building block was a di-anionic bidentate chelator at the silicon atom. In solution, the complexes were involved in rapid coordination–dissociation equilibria between the pentacoordinate Si complex (e.g., **(Aib)SiMe_2_-NMI**) and its constituents NMI and a five-membered silaheterocycle (e.g., **(Aib)SiMe_2_**), as shown by ^29^Si NMR spectroscopy. The energetics of the Lewis acid-base adduct formation and the competing solvation of the NMI molecule by chloroform were assessed with the aid of computational methods. In CDCl_3_ solution, deuteration of the silaheterocycle NH group proceeded rapidly, with more than 50% conversion within two days. Upon cooling to −44 °C, the chloroform solvates of the adducts **(Aib)SiMe_2_-NMI** and **(Phg)SiMe_2_-NMI** crystallized from their parent solutions and allowed for their single-crystal X-ray diffraction analyses. In both cases, the Si atom was situated in a distorted trigonal bipyramidal coordination sphere with equatorial Si–C bonds and an equatorial Si–N bond (the one of the silaheterocycle). The axial positions were occupied by a carboxylate O atom of the silaheterocycle and the NMI ligand’s donor-*N*-atom.

## 1. Introduction

**Figure 1:**
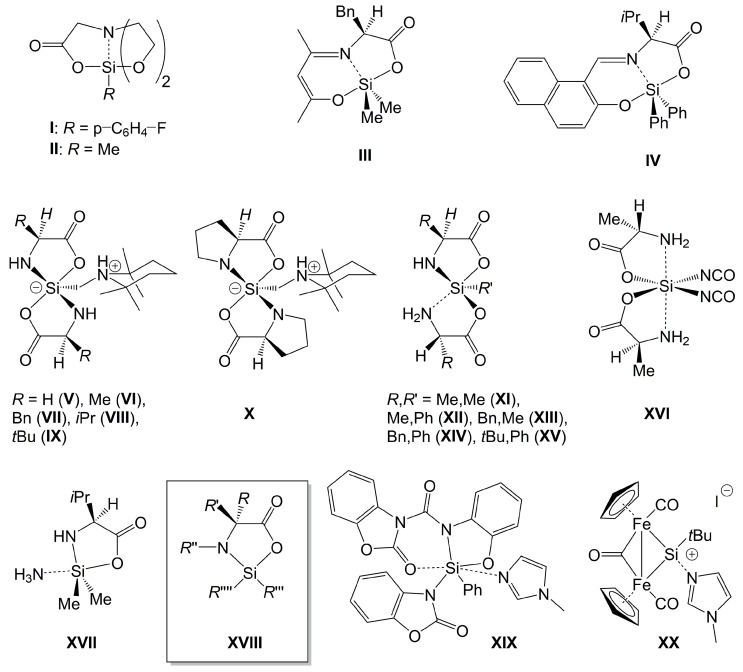
Examples of compounds which feature α-amino acid motifs in silatranes (**I**,**II**) and in Schiff base complexes of silicon (**III**,**IV**), overview of crystallographically characterized Si-compounds with mono- and/or dianionic α-amino acid anions as *O*,*N*-bidentate chelators (**V**–**XVII**). (Related silacyclic motifs with tetracoordinate Si atom, **XVIII**, with *R*,*R*′,*R*″ = H, hydrocarbyl, have not yet been confirmed crystallographically.) Selected examples of NMI adducts of silicon compounds: **XIX** and **XX**. For clarity reasons, formally dative bonds (to additional charge neutral lone pair donors) are drawn as dashed lines.

The chemistry of hypercoordinate silicon compounds (i.e., compounds in which the Si atom has a coordination number greater than four) have attracted scientists’ interest for more than two centuries. In 1812, Davy described the synthesis of the ammonia adduct SiF_4_(NH_3_)_2_ [1]. Even though ammonia remained a scarcely encountered ligand in silicon coordination chemistry, various other *N*-donor molecules were found capable of binding to silanes with electronegative substituents and thus enhancing the Si coordination number, e.g., in the adduct SiCl_4_(pyridine)_2_ [2]. The use of chelating ligands enhanced the variety of *N*-donors ligands, which thus formed penta- or hexacoordinate Si-compounds, and various reviews (e.g., [3,4,5,6,7,8]) provide insights into the wealth of silicon coordination chemistry with additional *N*-donors or with various other ligands. In this context, α-amino acids represent a class of chelators with *N*-donor functionality, which should be suitable to bind to silicon in a chelating manner (with the formation of five-membered rings, a common motif in Si-coordination chemistry), to enhance the Si coordination number. In some classes of oligodentate *O*,*N*-chelators the α-amino acid motif is contained, e.g., in silatranes with a carboxylate moiety (such as **I** and **II** [9,10], Figure 1) and in amino acid-derived Schiff base complexes (such as **III** and **IV** [11,12]). As many α-amino acids themselves are available from the natural chiral pool, it is noteworthy that only a comparatively small number of silicon complexes with their mono- or di-anionic α-amino acid ligands have been crystallographically characterized so far (compounds **V**–**XVII**) [13,14,15,16].

In this rather limited portfolio, our recently reported compound **XVII** [16] takes on a prominent role as it represents the first crystallographically characterized ammonia adduct of an organosilicon compound (and, moreover, the first adduct of an α-amino acid derived silaheterocycle with a neutral Lewis base). So far, there is no crystallographic evidence for the isolation and structural characterization of silacycles with di-anionic chelators of the α-amino acid type which feature tetracoordinate silicon. Neither simple silacycles of type **XVIII** nor related spirocycles can be found in the CSD (a search in CSD 2023 version 5.44). Nonetheless, compounds such as (Phe)SiMe_2_ [17] or (His)SiMe_2_ [18] (with Phe and His being the di-anions of phenylalanine and histidine, respectively) were reported in the literature more than two decades ago as intermediates in reactions such as amide formation [17] and the functionalization of a trityl type resin [18]. Ammonia adduct **XVII** decomposes when dissolved in chloroform with dissociation of the Si–NH_3_ bond and formation of a wealth of decomposition products with tetracoordinate Si atoms [16]. Thus, we conclude that this adduct formation, which enhances the Si coordination number, stabilizes these particular five-membered silacycles and, vice versa, this stabilizing effect gave rise to the formation of such a rare ammonia adduct of an organosilicon compound. As the addition of stoichiometric amounts of ammonia to a chloroform solution is hard to accomplish, and the removal of solvent during sample preparation may be accompanied by loss of this volatile ligand, adduct formation with an alternative *N*-donor molecule may be considered for related studies. *N*-methylimidazole (NMI) represents such an alternative. In principle, NMI is known as a good *N*-donor moiety in Si coordination chemistry. With a variety of silicon halides, it induces ionic dissociation of Si–*X* (*X* = Cl, Br) bonds with formation of cationic Si-complexes. Structurally characterized examples thereof are known for compounds with tetracoordinate Si: [Me_3_Si(NMI)]^+^*X*^−^ (*X* = Cl [19], Br [20]), [PhMe_2_Si(NMI)]^+^Cl^−^ [21], [*R*_2_Si(NMI)_2_]^2+^ 2Br^−^ (*R*_2_ = Me_2_ [22], Et_2_ [23], –(CH_2_)_5_– [24]), with pentacoordinate Si: [*R*_2_Si(NMI)_3_]^2+^ 2Br^−^ (*R*_2_ = Et,H [23], Ph_2_ [23]) and with hexacoordinate Si: [Cl_2_(NMI)_3_SiCH_2_-]_2_^2+^ 2Cl^−^ [25], [*R*,*R*’Si(NMI)_4_]^2+^ 2Br^−^ (*R*,*R*’ = Me,H [23], Ph,H [23], Cl,Cl [26]), [*R*_2_Si(NMI)_4_]^2+^ 2Cl^−^ (*R*_2_ = Cl_2_ [26], H_2_ [26], –(CH_2_)_4_– [27]). Furthermore, some non-ionic hexacoordinate Si-NMI-complexes have been crystallographically confirmed (Si(o-C_6_H_4_O_2_)_2_(NMI)_2_ [28], **XIX** [29]), and an adduct of the composition MeSiCl_3_(NMI)_2_ is mentioned in the literature [30]. In addition to just binding to Si (as in the latter cases) or activating Si–*X* bonds (as in the former cases), NMI was found to activate Si–Si bonds and thus catalyze the cleavage of a variety of disilanes with formation of oligosilanes [30,31], and it stabilized a low-coordinate transition metal–silicon complex (**XX** [32]). Because of this prominent tendency of adduct formation with silicon compounds, NMI became our *N*-donor of choice for the following study.

## 2. Results and Discussion

### 2.1. Compounds Overview

**Scheme 1:**
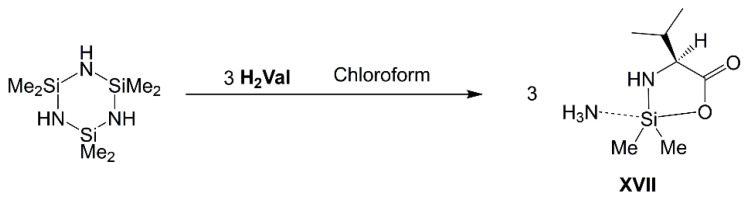
Reaction of the formation of compound **XVII** [16].

**Scheme 2:**
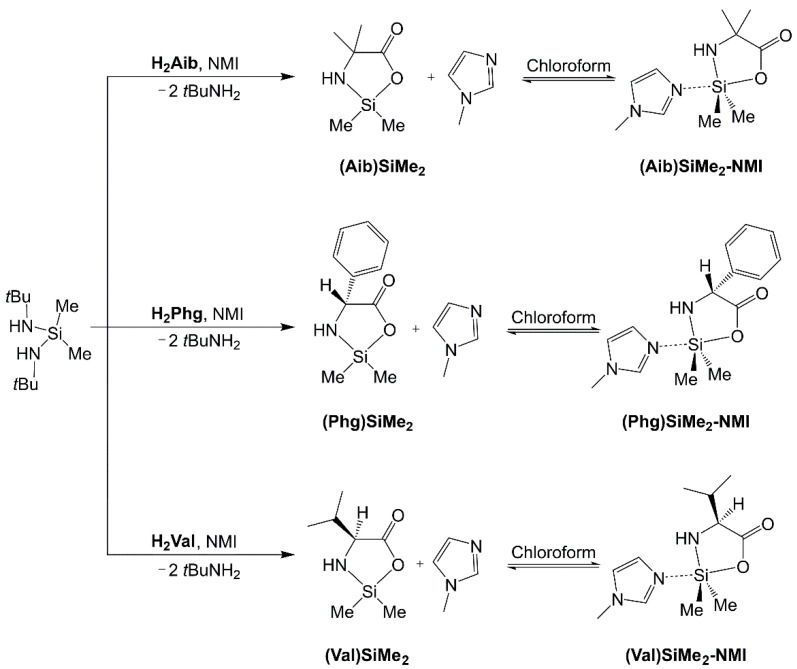
Syntheses of the compounds under investigation.

The previously reported compound **XVII** formed in a reaction of hexamethylcyclotrisilazane and L-valine [16]. The *N*-donor ligand of this adduct (i.e., ammonia) was the leaving group of the SiMe_2_-containing starting material (Scheme 1). In order to avoid the formation of ammonia-stabilized silaheterocycles as a potential competitive reaction, in favor of adduct formation with the target *N*-donor molecule NMI, we engaged a starting material with a sterically more demanding leaving group (*t*BuNH_2_), i.e., bis(tert-butylamino)dimethylsilane (Scheme 2). In chloroform solution, the three amino acids under investigation (α-aminoisobutyric acid **H_2_Aib**, D-phenylglycine **H_2_Phg** and L-valine **H_2_Val**) formed the respective target compound, i.e., the pentacoordinate Si complex **(Aib)SiMe_2_-NMI**, **(Phg)SiMe_2_-NMI** and **(Val)SiMe_2_-NMI**, respectively (cf. Section 2.3, NMR spectroscopic characterization). In two cases (**(Aib)SiMe_2_-NMI** and **(Phg)SiMe_2_-NMI**) we succeeded in obtaining these adducts as crystalline solids at −44 °C. Under these conditions, they crystallized as the chloroform solvates **(Aib)SiMe_2_-NMI · CHCl_3_** and **(Phg)SiMe_2_-NMI · 2CHCl_3_**, respectively, and their molecular structures were determined by single-crystal X-ray diffraction analysis. 

### 2.2. Single-Crystal X-ray Diffraction

**Figure 2:**
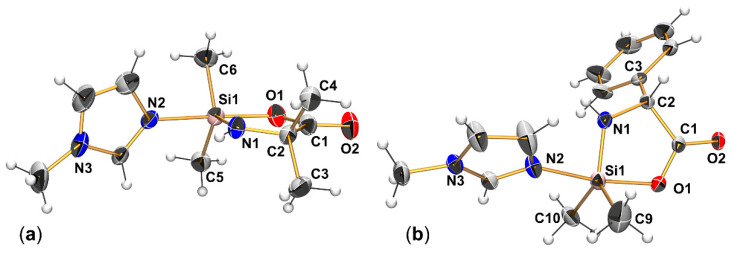
Molecular structures of (**a**) **(Aib)SiMe_2_-NMI** and (**b**) **(Phg)SiMe_2_-NMI** in the crystal structures of their chloroform solvates **(Aib)SiMe_2_-NMI · CHCl_3_** and **(Phg)SiMe_2_-NMI · 2CHCl_3_**, respectively (displacement ellipsoids drawn at the 30% probability level, selected atoms labeled). For clarity reasons, solvent molecules are omitted, and in the case of **(Aib)SiMe_2_-NMI**, only the predominant position of the disordered NMI group is shown.

The solvates **(Aib)SiMe_2_-NMI · CHCl_3_** and **(Phg)SiMe_2_-NMI · 2CHCl_3_**, which crystallized upon storage of the reaction mixture at −44 °C, could be isolated from the supernatant by decantation while cold, but these solids formed a melt at room temperature. In the case of **(Phg)SiMe_2_-NMI · 2CHCl_3_**, which crystallized in compact blocks, the use of an ice-cooled Petri dish was sufficient for crystal preparation for X-ray diffraction analysis. In the case of **(Aib)SiMe_2_-NMI · CHCl_3_**, which formed thin needles, the solid was allowed to melt at room temperature. A small amount of this melt was transferred into a glass capillary, which was then sealed and mounted on the goniometer. Upon slow cooling on the diffractometer, crystals of this solvate formed again, and a single-crystalline needle was located inside the capillary, which was suitable for X-ray diffraction analysis. The molecular structures of these NMI adducts are shown in Figure 2; selected bond lengths and angles are listed in Table 1, and selected parameters of the data collection and structure refinement are listed in Appendix A, Table A1.

In compounds **(Aib)SiMe_2_-NMI** and **(Phg)SiMe_2_-NMI**, the Si atom is accommodated in a distorted trigonal bipyramidal coordination sphere, which is set up by the Si–O bond and the formally dative Si–N bond to the additional *N*-donor ligand (NMI) in axial positions, while the Si–C bonds and the formally covalent Si–N bond to the di-anionic chelator reside in equatorial positions. Among the two NMI adducts and, moreover, with respect to the ammonia adduct **XVII**, the corresponding bond lengths of these molecules are similar to one another. Most noteworthy, even the Si1–N2 bonds to the additional *N*-donor ligand are in a very narrow range in spite of the different donor molecules and their N atoms’ hybridization (sp^2^ in NMI, sp^3^ in ammonia). Corresponding angles in the Si atoms’ coordination spheres are also within very narrow ranges, and the geometry index *τ*_5_ [33], which is derived therefrom, clearly points at the predominance of the trigonal bipyramidal shape (over square pyramidal) to similar degrees in the three cases. The deviation of the axial angle from linearity (by ca. 8–9 deg.) originates only in part from the bite angle of the amino acid di-anion (which is ca. 5 deg. below the right angle). The systematically “too small” C–Si–C angle (with respect to VSEPR) and the resultant repulsion of the *N*-donor ligand from the SiMe_2_ moiety can be regarded as making another contribution. Within the equatorial arrangement of one Si–N and two Si–C bonds, the latter (because of the lower electronegativity of C vs. N) should exert pronounced repulsion. Thus, it is a noteworthy observation that the Si coordination spheres of the adducts listed in Table 1 exhibit C–Si–C angles well below 120°. We interpret this “too small” C–Si–C angle as a result of pronounced “equatoriophilicity”. Whereas widening of the C–Si–C angle would agree with VSEPR and Bent’s rule [34] (i.e., the central atom’s bonds to the substituents with lower electronegativity feature pronounced s-orbital contributions, and thus favor a transition from sp^3^ toward sp^2^ or even sp hybrid orbitals), it would also shift the Si–C bonds into more axial positions and result in their being more *trans*-disposed to one another. Thus, we interpret the rather narrow C–Si–C angle as a result of avoidance of a *trans*-C–Si–C arrangement, which would shift the character of the Si–C bonds from two 2e2c-bonds to a 4e3c-bond system. A search in the CSD (CSD 2023 version 5.44) revealed that most of the pentacoordinate dimethylsilicon compounds of the type Me_2_Si(X,X′,X″) (with X,X′,X″ being atoms from the group N,O,F,Cl) exhibit C–Si–C angles below or close to 120°. Only some representants with a pronounced electron deficient Si atom (substituents with very high electronegativity and/or cationic complexes) exhibit C–Si–C angles well above 120°: [Me_2_SiF_3_]^−^ 124.4° [35], [Me_2_Si(OTf)(bipy)]^+^ (OTf = trifluoromethanesulfonate, bipy = 2,2′-bipyridyl) 128.5° [36] and [Me_2_Si(NMI)_3_]^2+^ 130.8° [19].

**Figure 3:**
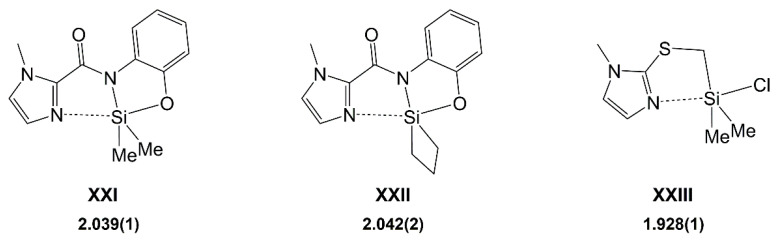
Examples of crystallographically characterized neutral pentacoordinate Si-compounds which feature a chelate-bound NMI derivative (NMI′) as an *N*-donor ligand in the trigonal bipyramidal Si-coordination sphere. Their Si–N(NMI′) bond lengths are given in Å.

The Si1–N2 bond lengths (to the NMI molecule) in **(Aib)SiMe_2_-NMI** and **(Phg)SiMe_2_-NMI** are slightly longer than the corresponding bond in the neutral hexacoordinate Si complex **XIX** (1.962(1) Å) [29] as well as in the ionic complex [SiPhH(NMI)_4_]^2+^ (1.959(4)–1.970(4) Å) [23], and markedly longer than in the ionic hexacoordinate Si-complex [SiCl_2_(NMI)_4_]^2+^, in which Si–N bond lengths are found in the range 1.876(3)–1.927(4) Å [26]. In related cationic Si complexes with a pentacoordinate Si atom, the axial Si–N(NMI) bonds are markedly longer than the equatorial ones, e.g., in [SiEtH(NMI)_3_]^2+^ (1.979(2), 1.952(2) Å vs. 1.817(2) Å) [23]. Whereas the axial Si–N(NMI) bond lengths are similar to those in hexacoordinate Si complexes, the equatorial ones are closer to Si–N(NMI) bonds in related cationic complexes with a tetracoordinate Si atom, e.g., in [SiEt_2_(NMI)_2_]^2+^ (1.796(2) and 1.798(2) Å) [23]. With respect to neutral pentacoordinate Si-complexes with NMI as an *N*-donor ligand, compounds in **(Aib)SiMe_2_-NMI** and **(Phg)SiMe_2_-NMI** are the first crystallographically characterized representatives. Some related motifs involve chelate-bound NMI derivatives in axial positions at the pentacoordinate Si atom (compounds **XXI** [37], **XXII** [38], **XXIII** [39], Figure 3). Comparison reveals that the Si–N(NMI) bond lengths of **(Aib)SiMe_2_-NMI** and **(Phg)SiMe_2_-NMI** are similar to the corresponding bonds in compounds **XXI** and **XXII**, which also feature *trans*-disposed O-atoms. The partial ionic dissociation of the Si–Cl bond in compound **XXIII** [39] can be interpreted as a contributor to the shorter Si–N bond, in accordance with the short Si–N(NMI) bonds in cationic hypercoordinate Si-NMI-complexes.

**Figure 4:**
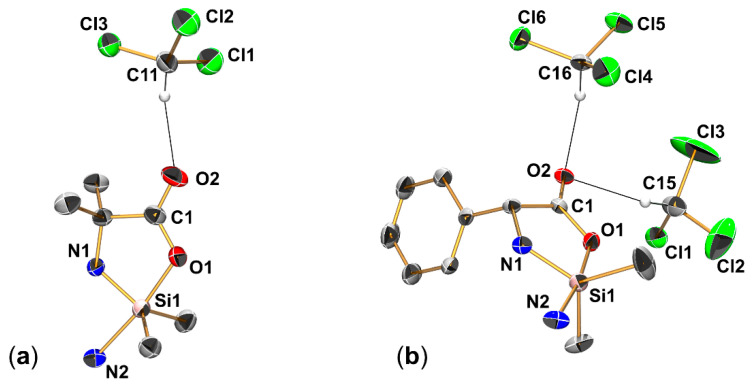
Selected parts of the molecular structures of (**a**) **(Aib)SiMe_2_-NMI** and (**b**) **(Phg)SiMe_2_-NMI** with O···H–CCl_3_ contacts in the crystal structures of their chloroform solvates **(Aib)SiMe_2_-NMI · CHCl_3_** and **(Phg)SiMe_2_-NMI · 2CHCl_3_**, respectively (displacement ellipsoids drawn at the 30% probability level, selected atoms labeled). For clarity reasons, only the predominant positions of the disordered chloroform molecules are shown. Parameters of the depicted H-contacts for (**a**) O2–C11 3.107(10) Å, O2–H–C11 153 deg.; for (**b**) O2–C15 3.267(11) Å, O2–H–C15 155 deg.; O2–C16 3.113(6) Å, O2–H–C16 149 deg.

In contrast to compound **XVII**, which crystallizes from chloroform without the inclusion of solvent of crystallization, both **(Aib)SiMe_2_-NMI** and **(Phg)SiMe_2_-NMI** crystallize as chloroform solvates, and in both cases the solvate molecules establish Cl_3_C–H···O contacts with the carbonyl O atom O2 of the amino acid moiety (Figure 4). Furthermore, in both cases the NMI moiety of an adjacent molecule forms two C–H···O2 contacts with a methyl C–H bond and the imidazole-C^2^–H bond involved in a chelate-like manner. (In the crystal structure of ammonia adduct **XVII**, the *N*-donor molecule, i.e., ammonia itself, serves as a hydrogen bridge donor toward the carbonyl O atom of an adjacent molecule.) The different degree of solvation of O2 (by one CHCl_3_ molecule in **(Aib)SiMe_2_-NMI** and by two CHCl_3_ molecules in **(Phg)SiMe_2_-NMI**) can be interpreted as a contributor to the longer Si1–O1 bond in the latter complex and, in response to this Si–O bond weakening, to the shorter Si1–N2 bond to the NMI moiety. This effect of chloroform solvation of the carbonyl O atom on the O–Si–N coordination axis will be elucidated further by computational analyses (cf. Section 2.4.2).

### 2.3. NMR Spectroscopic Analyses

**Figure 5:**
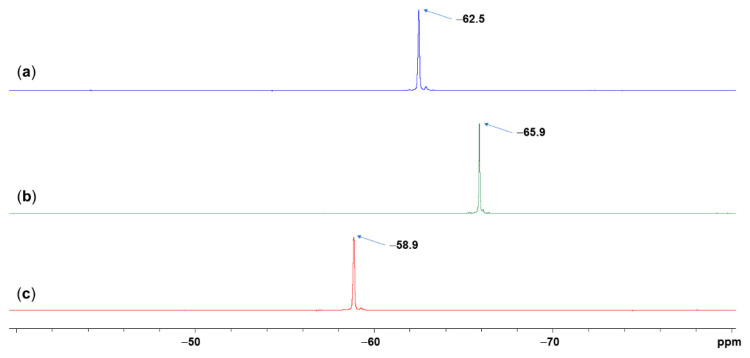
Selected parts of the ^29^Si{^1^H} INEPT NMR spectra of product solutions (**a**) **(Aib)SiMe_2_-NMI-4**, (**b**) **(Phg)SiMe_2_-NMI-4** and (**c**) **(Val)SiMe_2_-NMI-4** (cf. Table 2). The spectra are internally referenced to SiMe_4_ at 0 ppm.

**Figure 6:**
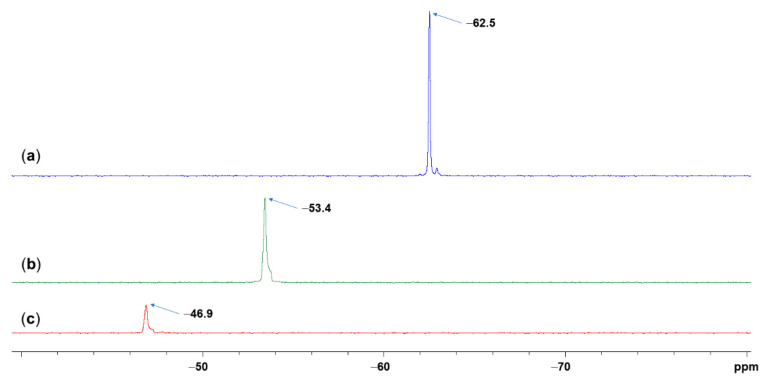
Selected parts of the ^29^Si{^1^H} INEPT NMR spectra of product solutions (**a**) **(Aib)SiMe_2_-NMI-4**, (**b**) **(Aib)SiMe_2_-NMI-2** and (**c**) **(Aib)SiMe_2_-NMI-4-dil** (cf. Table 2). The spectra are internally referenced to SiMe_4_ at 0 ppm.

As mentioned above, only two of the three adducts under investigation could be isolated as solids for single-crystal X-ray diffraction analyses. Isolation of the solids on a preparative scale was hampered by the good solubility of the compounds in the solvent used and their decomposition in the absence of NMI. Cold decantation and washing with chloroform afforded rather small amounts of these solids, which melted below room temperature and were not useful for solid state NMR spectroscopic characterization. In CDCl_3_ solution these compounds decomposed, as indicated by their ^29^Si{^1^H} NMR spectra, which exhibited many signals in addition to a broader signal, which can be assigned to the NMI adduct **(*Amac*)SiMe_2_-NMI** (*Amac* = Aib, Phg). (For these spectra, see Figures S1 and S2). Therefore, we characterized these compounds in their synthesis solution. For that purpose, the syntheses were performed on NMR scale in CDCl_3_ as the solvent. Table 2 lists the amounts of starting materials used in these experiments. Figure 5 shows the highfield section of the ^29^Si{^1^H} INEPT NMR spectra of the solutions **(*Amac*)SiMe_2_-NMI-4** (*Amac* = Aib, Phg, Val). These signals, which represent the main product of each solution (for the full spectra, see Figure S13 in the supporting information), emerge at ^29^Si NMR shifts characteristic of SiMe_2_-compounds with pentacoordinate silicon atoms. For example, compound **XVII** has a ^29^Si NMR shift of −75.5 ppm in the solid state [16], and compound **XXI** produces a signal at −59.2 ppm [37]. The position of the signal strongly depends on the stoichiometry of the starting materials used and on the degree of dilution, as shown for the series of **(Aib)SiMe_2_-NMI** batches in Figure 6. Thus, we conclude that these NMI adducts are part of dynamic equilibria, which involve compounds with tetracoordinate and compounds with a pentacoordinate Si atom (as indicated in Scheme 2). Both lowered NMI concentration (cf. batch **(Aib)SiMe_2_-NMI-2**) and lowered total concentration (cf. the diluted batch **(Aib)SiMe_2_-NMI-4-dil**) shift the equilibrium toward Si–N(NMI) bond dissociation and enhanced concentration of the compound with a tetracoordinate Si atom. 

**Figure 7:**
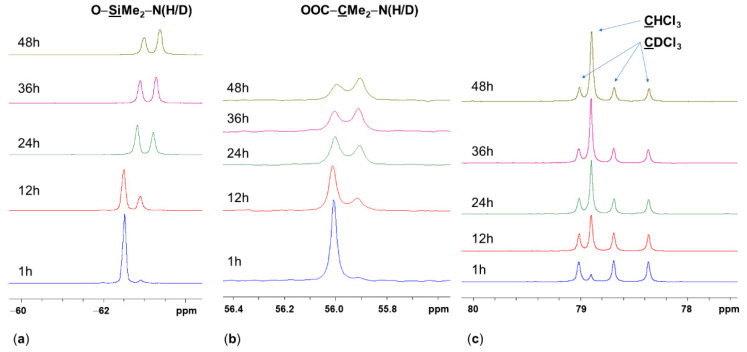
Selected parts of (**a**) the ^29^Si{^1^H} INEPT and (**b**,**c**) ^13^C{^1^H} NMR spectra of product solution **(Aib)SiMe_2_-NMI-4** at the indicated times of storage at room temperature. The spectra are internally referenced to SiMe_4_ at 0 ppm.

**Figure 8:**
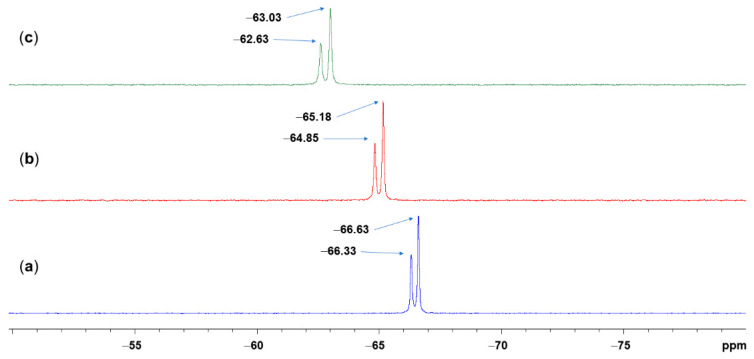
Selected parts of the ^29^Si{^1^H} INEPT NMR spectra of product solution **(Aib)SiMe_2_-NMI-4**, after 72 h of storage at room temperature, recorded at (**a**) 15 °C, (**b**) 20 °C and (**c**) 25 °C. The spectra are internally referenced to SiMe_4_ at 0 ppm.

In the spectra in Figure 5 and Figure 6, a small additional peak emerges slightly upfield shifted relative to each main signal. Time dependent ^29^Si NMR spectroscopic monitoring of sample **(Aib)SiMe_2_-NMI-4** (Figure 7a) revealed that this peak became the predominant signal in the course of two days. Simultaneously, the ^13^C NMR signal of this compound’s α-C atom (Figure 7b) exhibited a similar development of a new peak, and the solvent signal (Figure 7c) indicated the conversion of CDCl_3_ into CHCl_3_. Thus, the respective new signals in Figure 7a,b are assigned to the *N*-deuterated compound **(Aib)SiMe_2_-NMI-4**. In Figure 7a, it is evident that the positions of the signals exhibit slight variations. A temperature-dependent study of solution **(Aib)SiMe_2_-NMI-4** (spectra recorded at 15, 20, and 25 °C) revealed the strong temperature dependence of these ^29^Si NMR signals’ shifts and of the position of the equilibrium of NMI adduct formation and dissociation (Figure 8). Thus, we interpret the ^29^Si NMR shift variations in Figure 7a as an effect of the temperature variations in the NMR laboratory in the course of this time-dependent study.

As the NMR shifts of these compounds (both ^29^Si shifts as well as ^1^H and ^13^C NMR shifts) are highly dependent on concentration and temperature and, moreover, the solutions used in this study also contained the byproduct *t*BuNH_2_ in stoichiometric amounts, we do not report the NMR shifts observed as characteristic fingerprint features of these complexes. Instead, the sets of ^1^H, ^13^C{^1^H} and ^29^Si{^1^H} INEPT NMR spectra of the freshly prepared samples **(Aib)SiMe_2_-NMI-4**, **(Phg)SiMe_2_-NMI-4** and **(Val)SiMe_2_-NMI-4** (together with signal assignment) are provided as representative examples in the supporting information (Figures S3–S13).

### 2.4. Computational Analyses

#### 2.4.1. Calculation of ^29^Si NMR Shifts

As the solution NMR experiments (cf. Section 2.3) have indicated, the species **(*Amac*)SiMe_2_** with a tetracoordinate Si atom (*Amac* = generic expression for the di-anion of the respective α-amino acid used) and NMI are in a dynamic equilibrium with the NMI adduct **(*Amac*)SiMe_2_-NMI** (with a pentacoordinate Si atom). As the tetracoordinate Si-compounds **(*Amac*)SiMe_2_** are not stable (according to our previous experience with the decomposition of compound **XVII** in chloroform solution), and the compounds of the type **(*Amac*)SiMe_2_-NMI** of the current study were not accessible for solid state NMR investigations, we evaluated the ^29^Si NMR shifts of those species by computational methods. The knowledge of these boundaries (i.e., of the expected ^29^Si NMR shifts of the individual compounds with a tetra- and pentacoordinate Si atom) allows us to evaluate the position of the dynamic equilibrium under the conditions of this study. The ^29^Si NMR signal observed (at *δ*^29^Si_exp_) appears at the weighted chemical shift between the shifts of the two contributors **(*Amac*)SiMe_2_** and **(*Amac*)SiMe_2_-NMI**: *δ*^29^Si(observed) = *δ*^29^Si(**(*Amac*)SiMe_2_**)·*X*(**(*Amac*)SiMe_2_**) + *δ*^29^Si(**(*Amac*)SiMe_2_-NMI**)·*X*(**(*Amac*)SiMe_2_-NMI**), with *X* being the respective molar fractions. For that purpose, the three species **(*Amac*)SiMe_2_-CHCl_3_** and their respective NMI adducts **(*Amac*)SiMe_2_-NMI-CHCl_3_** as well as reference molecule SiMe_4_ were optimized at the same level of theory within a continuous solvent environment model (COSMO) for chloroform (We decided to use the CHCl_3_ solvated representatives for this purpose because further calculations, cf. Section 2.4.2, have shown that solvation of the carbonyl O atom by CHCl_3_ is energetically even more important than NMI-coordination at the Si atom). The calculated ^29^Si NMR shifts as well as the experimentally observed data are collated in Table 3. The combination of these experimentally and computationally obtained ^29^Si NMR shifts indicates that in the three samples under investigation, only 85–90% of the silicon compound are NMI-complexes and, in spite of the large excess of NMI used (four equivalents), more than 10% of the tetracoordinate Si-compound is contained in the equilibrium mixture. 

The experimental data are based on in situ monitoring of samples which contain rather high concentrations of the sample material (and thus the solvent used must be regarded as a stoichiometric reactant used in excess rather than having a very large excess of solvent). Furthermore, the samples contain another component (*t*BuNH_2_), which also has hydrogen bridge donor and acceptor qualities, which may thus compete with CHCl_3_ and NMI in hydrogen bond-forming reactions. Therefore, we investigated the energetics of some competitive donor–acceptor interactions in simplified systems [**(*Amac*)SiMe_2_** + NMI + CHCl_3_] with the aid of computational methods (Section 2.4.2).

#### 2.4.2. Evaluation of Structural Effects of NMI Coordination and CHCl_3_ Solvation

**Scheme 3:**
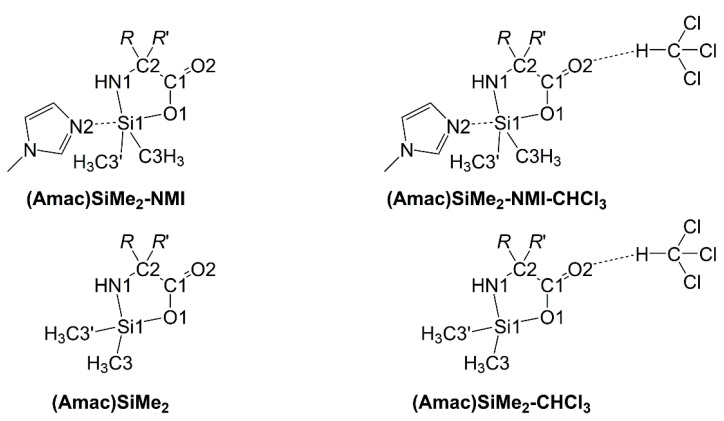
Atom numbering scheme for the comparison of selected bond lengths in the optimized molecular structures of **(*Amac*)SiMe_2_-NMI** and **(*Amac*)SiMe_2_** as well as their chloroform solvates **(*Amac*)SiMe_2_-NMI-CHCl_3_** and **(*Amac*)SiMe_2_-CHCl_3_** for *Amac* = Aib, Phg, Val (cf. Table 4 and Table 5).

For the current study, we investigated the systems [**(*Amac*)SiMe_2_** + NMI + CHCl_3_] as diluted solutions in excess solvent. For that purpose, the molecules of the silicon-containing species, the free NMI molecule, and the individual CHCl_3_ molecule (which was used for explicit solvation of particular hydrogen bond acceptor sites) as well as their adducts were optimized in a continuous solvent environment model (COSMO). The optimized molecular structures of the optimized silicon compounds (Scheme 3) as well as those of NMI and CHCl_3_ are shown in the supporting information (Figures S14–S28). Some selected structural features of the four different types of silicon compounds are listed in Table 4 and Table 5.

Both Table 4 and Table 5 mirror the effect of chloroform solvation of the carbonyl O atom on the bond lengths in the COO-motif and the Si coordination sphere in their last column. The ØΔ listed there are the average values (from the three sets of couples of compounds) of bond lengthening (+) or shortening (−) upon chloroform solvation. In the tetracoordinate Si-compounds **(*Amac*)SiMe_2_** (Table 4), solvation of the carbonyl O atom results in Cl_3_C–H···O hydrogen bonds of ca. 2.05–2.11 Å H···O separation. This causes a slight lengthening of the C=O bond (by 0.004 Å), shortening of the C–O bond (by 0.006 Å), and slight lengthening of the Si–O bond (by 0.004 Å). The effects on other bonds are less pronounced. In the corresponding NMI adducts with a pentacoordinate Si atom (Table 5), solvation of the carbonyl O atom is more pronounced (Cl_3_C–H···O hydrogen bonds of ca. 1.97–2.02 Å H···O separation). Even though the effect on the bond lengths in the carboxyl group is similar to that of the corresponding NMI-free tetracoordinate Si-compounds, the Si–O bond lengthening is noticeable more pronounced (0.011 Å). This Si–O bond lengthening is accompanied by an even more pronounced Si–N(NMI) bond shortening (by 0.015 Å). These observations indicate cooperative “solvation” of compounds **(*Amac*)SiMe_2_** by chloroform and NMI. While CHCl_3_ solvation induces pronounced Si–N(NMI) bond shortening, the presence of NMI at the Si atom allows for more pronounced solvation of the carbonyl O atom (shorter Cl_3_C–H···O hydrogen bonds). In this regard, this comparison is in accordance with the discussion of the molecular structures of solvates **(Aib)SiMe_2_-NMI · CHCl_3_** and **(Phg)SiMe_2_-NMI · 2CHCl_3_**, which deliver experimental support for longer Si–O and shorter Si–N(NMI) bonds in case of enhanced chloroform solvation of the carbonyl O atom. 

#### 2.4.3. Evaluation of Energetic Effects of NMI Coordination and CHCl_3_ Solvation

In addition to compounds **(*Amac*)SiMe_2_** and the different adducts or solvates **(*Amac*)SiMe_2_-NMI**, **(*Amac*)SiMe_2_-CHCl_3_**, and **(*Amac*)SiMe_2_-NMI-CHCl_3_** (for *Amac* = Aib, Phg, Val; cf. Scheme 3), the molecules NMI and CHCl_3_ as well as the hydrogen bonded adduct **NMI-CHCl_3_** were optimized at the same level of theory. From the single point energies of the optimized molecular structures we derived their Gibbs free energies at 293.15 K. Table 6 lists the differences in energies and in Gibbs free energies associated with the formation of the different kinds of adducts. 

The formation reactions of the NMI adducts **(*Amac*)SiMe_2_-NMI** out of the respective heterocycle **(*Amac*)SiMe_2_** and NMI are predicted to be exothermal (up to −11.9 kcal·mol^−1^) but endergonic at room temperature (up to 4.0 kcal·mol^−1^). The formation of the H-bonded solvates **(*Amac*)SiMe_2_-CHCl_3_** is less exothermal (up to −5.6 kcal·mol^−1^) and more endergonic (up to 8.3 kcal·mol^−1^) than NMI-Si-complex formation. Moreover, this solvate formation is energetically similar to NMI-CHCl_3_-solvation (exothermal, −5.1 kcal·mol^−1^, endergonic, 7.0 kcal·mol^−1^). Thus, coordination of NMI to the Si atom of **(*Amac*)SiMe_2_** should be slightly favored over the formation of an H-bonded adduct with the solvent. 

Note: The predicted endergonic character of the aforementioned reactions may originate from the assumption of isolated molecules (in a polar environment, COSMO), which is comparable to entropy effects in gas phase (a “polar gas phase”, though). However, experiments were carried out in the condensed phase. All of these reactions have in common a decrease of the number of molecules by 1 mol (from starting materials to products). Thus, the overestimation of endergonic character could be described as the effect of the loss of entropy of vaporization for 1 mol of molecules, which should be close to 6 kcal·mol^−1^ at 293.15 K for molecules without specific interactions (according to Trouton’s rule [40]). Taking this into consideration, the corrected Gibbs free energies should be closer to 0 kcal·mol^−1^ (slightly exergonic for the formation of **(*Amac*)SiMe_2_-NMI**, and, if still endergonic, only slightly endergonic for the formation of chloroform solvates **(*Amac*)SiMe_2_-CHCl_3_** and **NMI-CHCl_3_**.)

As in excess chloroform (used as the solvent), the non-solvated species **(*Amac*)SiMe_2_** and free NMI appear unlikely (in the latter case, experimental thermochemical studies revealed the exothermal dissolution of NMI in chloroform [41]); we consider the solvates **(*Amac*)SiMe_2_-CHCl_3_** and **NMI-CHCl_3_** as more relevant starting materials involved in further reactions such as the formation of compounds **(*Amac*)SiMe_2_-NMI-CHCl_3_**. Therefore, instead of the formation of **(*Amac*)SiMe_2_-NMI-CHCl_3_** out of the three individual components and **(*Amac*)SiMe_2_** + NMI + CHCl_3_ (associated with changes in energy up to −18.6 kcal·mol^−1^ and in Gibbs free energy up to 10.9 kcal·mol^−1^, which can thus be expected to be slightly exergonic upon correction by ca. 12 kcal·mol^−1^, i.e., the number of molecules changed by 2 mol in this case), we regard the reaction **(*Amac*)SiMe_2_-CHCl_3_** + **NMI-CHCl_3_** → **(*Amac*)SiMe_2_-NMI-CHCl_3_** + CHCl_3_ as more relevant. The changes in energy and Gibbs free energy associated with this reaction (in Table 6 with the suffix “eff.”, up to −7.9 and to −5.1 kcal·mol^−1^, respectively) are slightly exothermal and exergonic, respectively, and should reflect the thermodynamic foundation for the association–dissociation equilibria in these solutions in a more reasonable way. Nonetheless, this prediction is associated with a too-exergonic Gibbs free energy. As the solution ^29^Si NMR experiments have shown that both tetra- and pentacoordinate Si-complexes must coexist in an equilibrium in concentrations of the same order of magnitude, it should be closer to 0 kcal·mol^−1^. We attribute the deviations between the experiment and computational study to the more complex system in the real solutions. The latter involve further components (such as *t*BuNH_2_), which may contribute to entropy in a statistical manner, and they involve further possibilities of specific interactions (such as hydrogen bonding of other H-bond donor and acceptor moieties), which have not been considered in this study. For example, even in case of the simple molecule NMI, further solvation with chloroform (which exceeds the 1:1 adduct formation) may be considered to contribute. Rakipov et al. attributed the pronounced exothermal reaction of NMI and chloroform to the 1:2 solvate formation in chloroform-rich solution [41].

Apart from the deviation of the exergonic character of the reactions from the expected range of close to 0 kcal·mol^−1^, the energy values listed in Table 6 give rise to the conclusion that these systems exhibit cooperative adduct formation. The simultaneous binding of both chloroform (to the carbonyl O atom) and NMI (to the Si atom) at the **(*Amac*)SiMe_2_** moiety with the formation of **(*Amac*)SiMe_2_-NMI-CHCl_3_** is thermodynamically favored over the individual binding of NMI and CHCl_3_ with formation of **(*Amac*)SiMe_2_-NMI** and **(*Amac*)SiMe_2_-CHCl_3_**. This is reflected in Table 6 by the entries with the suffix “coop.”, which represent the energetics of the reaction **(*Amac*)SiMe_2_-NMI** + **(*Amac*)SiMe_2_-CHCl_3_** → **(*Amac*)SiMe_2_-NMI-CHCl_3_** + **(*Amac*)SiMe_2_**. In addition to the cooperative effect of the binding of NMI and CHCl_3_ to the **(*Amac*)SiMe_2_** moiety on the molecular structures of **(*Amac*)SiMe_2_-NMI-CHCl_3_** (shortening of Cl_3_C–H···O H-bonds by NMI-coordination at Si, and shortening of Si–N(NMI) bonds by chloroform solvation of the carbonyl O atom), this mutual binding makes a thermodynamic contribution to the stabilization of these pentacoordinate silicon complexes. 

## 3. Materials and Methods

### 3.1. General Considerations

The starting material (*t*BuNH)_2_SiMe_2_ was prepared according to a published method [42]. The amino acids α-aminoisobutyric acid (Roth, Karlsruhe, Germany, ≥97%), D-phenylglycine, and L-valine (Merck, Darmstadt, Germany, ≥99%) were commercially available and were used without further purification. (Note: For the chiral amino acids, phenylglycine, and valine, the choice of the respective enantiomer was made by the availability of the starting materials from previous studies.) *N*-Methylimidazole (Sigma-Aldrich, Steinheim, Germany, ≥99%), chloroform, stabilized with amylenes (Honeywell, Seelze, Germany, ≥99.5%) and CDCl_3_ (Deutero, Kastellaun, Germany, 99.8%) were stored over activated molecular sieves (3 Å) for at least 7 days and used without further purification. All reactions were carried out under an atmosphere of dry argon utilizing standard Schlenk techniques. Solution NMR spectra (^1^H, ^13^C, ^29^Si) (cf. Figures S1–S13 in the supporting information) were recorded on Bruker Avance III 500 MHz and Bruker Nanobay 400 MHz spectrometers. ^1^H, ^13^C and ^29^Si chemical shifts are reported relative to Me_4_Si (0 ppm) as internal reference. (Note: The referencing of ^1^H and ^13^C chemical shifts against solvent signals is not useful in these systems which contain large amounts of strong hydrogen bond acceptors, e.g., NMI. Such components may influence the ^1^H and ^13^C chemical shift of chloroform noticeably. For instance, in a ^1^H NMR spectrum of a solution of NMI in TMS-containing CDCl_3_, which was recorded for a purity check of the NMI used, the residual CHCl_3_ signal emerged at 7.37 ppm rather than at 7.26 ppm in neat CDCl_3_.) For single-crystal X-ray diffraction analysis of **(Phg)SiMe_2_-NMI · 2CHCl_3_** a crystal was selected under an inert oil on an ice-cooled Petri dish, mounted on a glass capillary and instantly moved to the cold nitrogen stream of the diffractometer. In the case of **(Aib)SiMe_2_-NMI · CHCl_3_**, the compound was allowed to melt at room temperature, whereupon a small amount of the melt was transferred into a glass capillary, which was then sealed, mounted on the goniometer, and slowly cooled to allow for the re-crystallization of the compound inside the capillary on the diffractometer. (The crystallization procedure was similar to our approach of crystallizing chlorosilanes for X-ray diffraction analyses [43].) Diffraction data were collected on a Stoe IPDS-2 diffractometer (STOE, Darmstadt, Germany) using Mo Kα-radiation. Data integration and absorption correction were performed with the STOE software XArea (version 1.75) and XShape (version 2.17), respectively. The structures were solved using SHELXT [44] and refined with the full-matrix least-squares methods of *F*^2^ against all reflections with SHELXL-2018/3 [45,46]. All non-hydrogen atoms were anisotropically refined, and hydrogen atoms were isotropically refined in an idealized position (riding model). For details of data collection and refinement see Appendix A, Table A1. Graphics of molecular structures were generated with ORTEP-3 [47,48] and POV-Ray 3.7 [49]. 

The geometry optimizations were carried out with ORCA 5.0.3 [50] using the restricted PBE0 functional with relativistically recontracted Karlsruhe basis sets ZORA-def2-TZVPP [51,52] for all atoms, the scalar relativistic ZORA Hamiltonian [53,54], atom-pairwise dispersion correction with the Becke–Johnson damping scheme (D3BJ) [55,56], and COSMO solvation (CHCl_3_, ε = 4.8, rsolv = 3.17). Very-TightSCF and slowconv options were applied and the DEFGRID3 was used with a radial integration accuracy of 10 for Si for all calculations. Calculations were started from the molecular structures obtained by single-crystal X-ray diffraction analysis. Numerical frequency calculations were performed to prove convergence at the local minimum after geometry optimization and to obtain the Gibbs free energy (293.15 K). NMR calculations were carried out with ORCA 5.0.3 at the same level of theory as mentioned above. Graphics were generated using ChemCraft [57]. 

### 3.2. Synthesis and Characterization

Compound **(Aib)SiMe_2_-NMI**: In a small Schlenk tube, equipped with a magnetic stirring bar, α-amino isobutyric acid (**H_2_Aib**, 295 mg, 2.86 mmol) was evacuated and then set under an argon atmosphere. Upon addition of chloroform (2 mL) and NMI (0.99 g, 12.0 mmol) the mixture was cooled in an ice bath. To the cold mixture, the aminosilane (*t*BuNH)_2_SiMe_2_ (623 mg, 3.08 mmol) was added with a syringe, and stirring at 0 °C was continued for 6 h. (During that time, the amino acid had dissolved, and a clear solution was obtained.) Thereafter, the solution was immediately placed in a freezer (at −44 °C), where crystalline needles of the product formed over the course of some days. Upon the transfer of the Schlenk tubes into a cold bath (*n*-decane, cooled with dry ice, ca. −30 °C), the supernatant was removed with a syringe; the crystals were washed with a small amount of cold chloroform (ca. 1 mL, also cooled to −30 °C prior to use) and briefly dried in vacuum while cold. The product was then stored in a small ice bath, which was allowed to attain room temperature over the course of 1 h. Melting of the crystalline product was observed at ca. 15 °C water bath temperature. Yield: 464 mg, 1.29 mmol, 45% (yield is based on the composition **(Aib)SiMe_2_-NMI · CHCl_3_**, as found in the single-crystal X-ray diffraction analysis. According to ^29^Si NMR spectroscopy, the compound undergoes decomposition when dissolved in CDCl_3_ (cf. Figure S1 in the supporting information). 

Compound **(Phg)SiMe_2_-NMI**: The same synthesis and work-up procedure applies (see Compound **(Aib)SiMe_2_-NMI**); amounts of compounds used: D-phenylglycine, 301 mg, 1.99 mmol; NMI, 0.68 g, 8.28 mmol; (*t*BuNH)_2_SiMe_2_, 436 mg, 2.15 mmol; chloroform for synthesis and washing, 2 mL and 1 mL, respectively. Yield: 291 mg, 0.55 mmol, 28%. The initially crystalline product melted at ca. 15 °C. Calculation of the yield is based on the composition **(Phg)SiMe_2_-NMI · 2CHCl_3_**, as found in the single-crystal X-ray diffraction analysis. According to ^29^Si NMR spectroscopy, the compound undergoes decomposition when dissolved in CDCl_3_ (cf. Figure S2 in the supporting information). 

Compound **(Val)SiMe_2_-NMI**: The same synthesis and work-up procedure applies (see Compound **(Aib)SiMe_2_-NMI**); amounts of compounds used: L-valine, 291 mg, 2.48 mmol; NMI, 0.82 g, 9.99 mmol; (*t*BuNH)_2_SiMe_2_, 551 mg, 2.72 mmol; CHCl_3_, 2 mL. Even upon storage at −44 °C for two weeks and cycles of temperature change between freezers at −44 °C and −24 °C, this compound did not crystallize.

For further characterization of compounds **(*Amac*)SiMe_2_-NMI** (*Amac* = Aib, Phg, Val), solution samples were synthesized in CDCl_3_ and investigated with ^1^H, ^13^C{^1^H} and ^29^Si{^1^H} INEPT NMR spectroscopy in situ. The amounts of starting materials used are listed in Table 2. The same synthesis procedure (as listed above for **(Aib)SiMe_2_-NMI**) applies. For NMR spectroscopic data of these samples, see Figures S3–S13 in the supporting information.

## 4. Conclusions

To date, the solvates **(Aib)SiMe_2_-NMI · CHCl_3_** and **(Phg)SiMe_2_-NMI · 2CHCl_3_** represent the first crystallographically characterized neutral Si-NMI-adducts with a pentacoordinate silicon atom. In chloroform solution, compounds of the type **(*Amac*)SiMe_2_-NMI** (*Amac* = di-anionic moiety of an α-amino acid, in our study *Amac* = Aib, Phg, Val) are involved in association–dissociation equilibria. As none of the tetracoordinate silicon compounds of type **(*Amac*)Si*RR’*** (*Amac* = any di-anionic moiety of an α-amino acid, *R*,*R’* = any kind of substituent) has been characterized crystallographically yet (according to a search in CSD 2023 version 5.44), and literature reports allow the conclusion that these species are relevant as reaction intermediates [17,18], the herein reported NMI adducts indicate adduct formation with a Lewis base as a tool to stabilize these compounds and to isolate them, which may become useful for their deliberate application as starting materials in further reactions. (In this regard, the SiMe_2_ functionalization as a special kind of silylation may be of particular interest for amide formation. Recent studies have shown that methyltrimethoxysilane can be useful as a tool in direct amidation of carboxylic acids [58], and triaryl silanols may act as catalysts in amidation reactions [59]). Our crystallographic and computational investigation of chloroform solvates of these NMI adducts highlights the role of the presence of an H-bond donor at the carbonyl atom of the α-amino acid moiety. It enhances the binding of the additional Lewis base to the Si atom and thus aids the Lewis base stabilization of compounds **(*Amac*)Si*RR’***. Both NMI and chloroform bind to **(*Amac*)SiMe_2_** (*Amac* = Aib, Phg, Val) in a cooperative manner. This general principle (Lewis base and H-bond donor binding to Si and carbonyl-O atom respectively) is also true for the previously reported ammonia adduct (Val)SiMe_2_-NH_3_ (compound **XVII**), in which the Lewis base (NH_3_) also serves as an H-bond donor to an adjacent molecule in the solid state. The current study serves as a pioneer: The principle of stabilization and isolation of compounds **(*Amac*)Si*RR’*** by mutual stabilization with lone pair donor atoms at Si and H-bond donors at the carbonyl group is yet to be explored, and various potential fields of application for the compounds derived therefrom can be found in organic syntheses. 

## Figures and Tables

**Table 1:** Selected bond lengths (Å) and angles (deg.) in compounds **(Aib)SiMe_2_-NMI** and **(Phg)SiMe_2_-NMI** in the crystal structures of their chloroform solvates (as well as corresponding parameters of compound **XVII** [16]).

	(Aib)SiMe_2_-NMI	(Phg)SiMe_2_-NMI	XVII
Si1–O1	1.852(2)	1.873(2)	1.877(1)
Si1–N1	1.713(2)	1.716(3)	1.717(2)
Si1–N2	2.036(2)	2.011(2)	2.014(2)
Si1–C ^1^	1.864(3)	1.859(4)	1.870(2)
	1.867(3)	1.873(3)	1.876(2)
C1–O1	1.291(3)	1.290(3)	1.302(2)
C1–O2	1.227(3)	1.223(3)	1.226(2)
O1-Si1-N1	85.25(9)	84.69(10)	84.93(5)
O1-Si1-N2	172.33(16)	171.27(10)	171.08(6)
C-Si1-C ^1^	113.92(14)	115.2(3)	113.44(9)
N1-Si1-C ^1^	122.69(13)	120.98(16)	121.93(8)
	123.38(12)	123.8(2)	124.63(8)
*τ*_5_	0.816	0.791	0.774

^1^ Because of the different individual labels of the Si-bound C atoms, a generic “C” is used here, and groups of relevant bond lengths or angles are listed without differentiation between the two Si-bound C atoms.

**Table 2:** Amounts of starting materials (mg, mmol) used for the in situ characterization of compounds **(Aib)SiMe_2_-NMI**, **(Phg)SiMe_2_-NMI** and **(Val)SiMe_2_-NMI** in CDCl_3_ solution. (For each batch, 2.0 mL of CDCl_3_ were used).

	Amino Acid	(*t*BuNH)_2_SiMe_2_ ^1^	NMI
**(Aib)SiMe_2_-NMI-2**	300, 2.90	621, 3.07	504, 6.10
**(Aib)SiMe_2_-NMI-4**	300, 2.90	625, 3.09	1002, 12.20
**(Aib)SiMe_2_-NMI-4-dil ^2^**	300, 2.90	625, 3.09	1002, 12.20
**(Phg)SiMe_2_-NMI-4**	438, 2.90	633, 3.13	1005, 12.24
**(Val)SiMe_2_-NMI-4**	340, 2.90	629, 3.11	1012, 12.33

^1^ With respect to the 1:1 stoichiometry of the reaction of the respective amino acid and (*t*BuNH)_2_SiMe_2_, as shown in Scheme 2, slight excess of the silane (*t*BuNH)_2_SiMe_2_ was used to achieve complete conversion of the amino acid (which has poor solubility in chloroform) within a few hours. ^2^ A sample of 0.3 mL of batch **(Aib)SiMe_2_-NMI-4** was diluted with 0.3 mL of CDCl_3_.

**Table 3:** Calculated ^29^Si NMR shifts of species **(*Amac*)SiMe_2_-CHCl_3_** and **(*Amac*)SiMe_2_-NMI-CHCl_3_**), experimentally observed chemical shifts of equilibrium mixtures (cf. Figure 5) and estimated molar fraction *X* of the pentacoordinate Si complex **(*Amac*)SiMe_2_-NMI-CHCl_3_)** in this equilibrium.

(Amac)	δ^29^Si_calc_((Amac)SiMe_2_-CHCl_3_)	δ^29^Si_calc_((Amac)SiMe_2_-NMI-CHCl_3_)	δ^29^Si_exp_	X((Amac)SiMe_2_-NMI-CHCl_3_)
(Aib)	+25.0	−77.6	−62.5	0.853
(Phg)	+29.8	−77.1	−65.9	0.895
(Val)	+32.6	−74.6	−58.9	0.854

**Table 4:** Comparison of selected bond lengths (Å) in the optimized molecular structures of the tetracoordinate Si-compounds **(*Amac*)SiMe_2_** and their chloroform solvates **(*Amac*)SiMe_2_-CHCl_3_**) for *Amac* = Aib, Phg, Val. (ØΔ is the average bond length change upon solvation with chloroform. For labels of relevant atoms, cf. Scheme 3).

	(Aib)SiMe_2_	(Aib)SiMe_2_-CHCl_3_	(Phg)SiMe_2_	(Phg)SiMe_2_-CHCl_3_	(Val)SiMe_2_	(Val)SiMe_2_-CHCl_3_	ØΔ
C1–O2	1.201	1.205	1.199	1.202	1.201	1.205	+0.004
C1–O1	1.337	1.330	1.335	1.330	1.340	1.333	−0.006
C1–C2	1.534	1.533	1.536	1.533	1.526	1.524	−0.002
Si1–O1	1.704	1.706	1.704	1.708	1.702	1.707	+0.004
Si1–N1	1.712	1.710	1.711	1.710	1.714	1.712	−0.002
Si1–C3/3′	1.850	1.849	1.850	1.849	1.849	1.848	−0.001
	1.853	1.852	1.851	1.849	1.854	1.852	
Cl_3_CH···O2	-	2.057	-	2.107	-	2.051	-

**Table 5:** Comparison of selected atom distances (Å) in the optimized molecular structures of the pentacoordinate Si-compounds **(*Amac*)SiMe_2_-NMI** and their chloroform solvates **(*Amac*)SiMe_2_-NMI-CHCl_3_**) for *Amac* = Aib, Phg, Val. (ØΔ is the average bond length change upon solvation with chloroform. For labels of relevant atoms, cf. Scheme 3).

	(Aib)SiMe_2_-NMI	(Aib)SiMe_2_-NMI-CHCl_3_	(Phg)SiMe_2_-NMI	(Phg)SiMe_2_-NMI-CHCl_3_	(Val)SiMe_2_-NMI	(Val)SiMe_2_-NMI-CHCl_3_	ØΔ
C1–O2	1.215	1.220	1.213	1.218	1.215	1.220	+0.005
C1–O1	1.301	1.295	1.299	1.293	1.304	1.297	−0.006
C1–C2	1.529	1.528	1.533	1.529	1.523	1.521	−0.002
Si1–O1	1.820	1.828	1.825	1.835	1.815	1.830	+0.011
Si1–N1	1.728	1.728	1.732	1.731	1.731	1.734	+0.001
Si1–N2	2.079	2.061	2.067	2.055	2.077	2.063	−0.015
Si1–C3/3′	1.878	1.878	1.878	1.876	1.878	1.876	−0.001
	1.880	1.879	1.879	1.877	1.879	1.879	
Cl_3_CH···O2 ^1^	-	1.966 (0.091)	-	2.017 (0.090)	-	1.984 (0.067)	-

^1^ The values in parentheses represent the shortening of this H···O contact (in Å) with respect to the corresponding NMI-free compound **(*Amac*)SiMe_2_-CHCl_3_**, cf. Table 4.

**Table 6:** Calculated relative energies (and Gibbs free energies in parentheses) of adducts **(*Amac*)SiMe_2_-NMI**, **(*Amac*)SiMe_2_-CHCl_3_**, and **(*Amac*)SiMe_2_-NMI-CHCl_3_** (in kcal·mol^−1^) with respect to the corresponding energies of the individual sets of constituents **(*Amac*)SiMe_2_** + NMI, **(*Amac*)SiMe_2_** + CHCl_3_, and **(*Amac*)SiMe_2_** + NMI + CHCl_3_.

(Amac)	(Amac)SiMe_2_-NMI	(Amac)SiMe_2_-CHCl_3_	(Amac)SiMe_2_-NMI-CHCl_3_	(Amac)SiMe_2_-NMI-CHCl_3_ Eff. ^1^	(Amac)SiMe_2_-NMI-CHCl_3_ Coop. ^2^
**(Aib)**	−10.9 (3.5)	−4.9 (7.7)	−16.8 (10.9)	−6.8 (−3.8)	−1.0 (−0.3)
**(Phg)**	−11.9 (3.2)	−5.6 (7.7)	−18.6 (10.2)	−7.9 (−4.4)	−1.1 (−0.7)
**(Val)**	−11.1 (4.0)	−5.3 (8.3)	−17.5 (10.2)	−7.0 (−5.1)	−1.1 (−2.1)

^1^ Adduct formation NMI + CHCl_3_ → **NMI-CHCl_3_** is exothermal by −5.1 kcal·mol^−1^ and endergonic by 7.0 kcal·mol^−1^. These values are similar to those of the formation of **(*Amac*)SiMe_2_-CHCl_3_**. In a solvent environment of excess chloroform, we consider the effective formation (eff.) of **(*Amac*)SiMe_2_-NMI-CHCl_3_** along the following reaction more relevant: **(*Amac*)SiMe_2_-CHCl_3_** + **NMI-CHCl_3_ → (*Amac*)SiMe_2_-NMI-CHCl_3_** + CHCl_3_. ^2^ The suffix (coop.) indicates the energy associated with the cooperation of NMI and CHCl_3_ in simultaneous binding to **(*Amac*)SiMe_2_**. This energy is representative of the reaction **(*Amac*)SiMe_2_-NMI** + **(*Amac*)SiMe_2_-CHCl_3_ → (*Amac*)SiMe_2_-NMI-CHCl_3_** + **(*Amac*)SiMe_2_**.

## Data Availability

CCDC 2306931 (**(Aib)SiMe_2_-NMI · CHCl_3_**) and 2306932 (**(Phg)SiMe_2_-NMI · 2CHCl_3_**) contain the supplementary crystal data for this article. These data can be obtained free of charge from the Cambridge Crystallographic Data Centre via https://www.ccdc.cam.ac.uk/structures/ (accessed on 9 November 2023).

## Supplementary Materials

The following supporting information can be downloaded at: https://www.mdpi.com/article/10.3390/molecules28237816/s1, Crystallographic data for the compounds reported in this paper (in CIF format) and a document containing the following: NMR spectra (^1^H, ^13^C{^1^H}, ^29^Si{^1^H}) of the compounds **(*Amac*)SiMe_2_-NMI** (*Amac* = Aib, Phg, Val) (Figures S1–S13); graphics of optimized molecular structures and total energies (Figures S14–S29) as well as atomic coordinates (Tables S1–S16) of compounds **(*Amac*)SiMe_2_, (*Amac*)SiMe_2_-CHCl_3_, (*Amac*)SiMe_2_-NMI, (*Amac*)SiMe_2_-NMI-CHCl_3_** (*Amac* = Aib, Phg, Val), NMI, CHCl_3_, NMI-CHCl_3_ as well as SiMe_4_.

## Appendix A

**Table A1:** Crystallographic data from data collection and refinement for **(Aib)SiMe_2_-NMI · CHCl_3_** and **(Phg)SiMe_2_-NMI · 2CHCl_3_**.

Parameter	(Aib)SiMe_2_-NMI · CHCl_3_ ^1^	(Phg)SiMe_2_-NMI · 2CHCl_3_ ^2^
Formula	C_11_H_20_Cl_3_N_3_O_2_Si	C_16_H_21_Cl_6_N_3_O_2_Si
*M*_r_	360.74	528.15
*T* (K)	220(2)	180(2)
*λ* (Å)	0.71073	0.71073
Crystal system	monoclinic	orthorhombic
Space group	*P*2_1_/*n*	*P*2_1_2_1_2_1_ ^3^
*a* (Å)	8.2985(3)	10.1442(2)
*b* (Å)	11.9564(2)	15.5169(2)
*c* (Å)	18.3551(6)	15.9743(2)
*β* (°)	96.346(3)	90
*V* (Å^3^)	1810.04(9)	2514.46(7)
*Z*	4	4
*ρ*_calc_ (g·cm^−1^)	1.32	1.40
*μ*_MoKα_ (mm^−1^)	0.6	0.7
*F(000)*	752	1080
*θ*_max_(°), *R*_int_	26.0, 0.0507	28.0, 0.0446
Completeness	99.8%	99.9%
Reflns collected	26,805	65,042
Reflns unique	3543	6075
Restraints	165	206
Parameters	280	385
GoF	1.160	1.097
*R*1, *wR*2 [*I*>2*σ*(*I*)]	0.0472, 0.1185	0.0387, 0.0919
*R*1, *wR*2 (all data)	0.0562, 0.1224	0.0458, 0.0963
Largest peak/hole (e·Å^−3^)	0.19, −0.19	0.29, −0.18

^1^ In this crystal structure, the chloroform molecule as well as the NMI-ligand suffer disorder. The former was refined in three positions (with site occupancies 0.521(14), 0.365(10), 0.112(12)). As the NMI molecule is located near an adjacent chloroform site, its disorder is influenced by the former disorder of the molecule of solvent of crystallization. It was refined over two sites, with site occupancies 0.635(10), 0.365(10). ^2^ In this crystal structure, the two chloroform molecules suffer disorder. Each of them was refined over three positions, with sets of site occupancies of 0.414(4), 0.239(4), 0.347(4) and 0.530(4), 0.264(4), 0.206(4). ^3^ For this chiral structure, the absolute structure parameter was refined to *χ*_Flack_ = 0.008(17).
