# Supplementary material for: Lewis Acid-Base Adducts of α-Amino Acid-Derived Silaheterocycles and N-Methylimidazole
Source: Molecules. 2023 Nov 28;28(23):7816. doi: 10.3390/molecules28237816 (PMC10708346; doi:10.3390/molecules28237816)

checkCIF/PLATON report

Structure factors have been supplied for datablock(s) as097b_01, as102b_01

THIS REPORT IS FOR GUIDANCE ONLY. IF USED AS PART OF A REVIEW PROCEDURE FOR PUBLICATION, IT SHOULD NOT REPLACE THE EXPERTISE OF AN EXPERIENCED CRYSTALLOGRAPHIC REFEREE.

No syntax errors found.   CIF dictionary   Interpreting this report

Datablock: as102b_01

Bond precision: C-C = 0.0037 A   Wavelength=0.71073

Cell:	a=8.2985(3)	b=11.9564(2)	c=18.3551(6)
	alpha=90	beta=96.346(3)	gamma=90
Temperature:	220 K		

	Calculated	Reported
Volume	1810.04(9)	1810.04(9)
Space group	P 21/n	P 21/n
Hall group	-P 2yn	-P 2yn
Moiety formula	C10 H19 N3 O2 Si, C H Cl3	C10 H19 N3 O2 Si, C H Cl3
Sum formula	C11 H20 Cl3 N3 O2 Si	C11 H20 Cl3 N3 O2 Si
Mr	360.62	360.74
Dx,g cm-3	1.323	1.324
Z	4	4
Mu (mm-1)	0.576	0.576
F000	751.8	752.0
F000'	754.07	
h,k,lmax	10,14,22	10,14,22
Nref	3551	3543
Tmin,Tmax	0.506,0.562	0.173,0.609
Tmin'	0.056	

Correction method= # Reported T Limits: Tmin=0.173 Tmax=0.609
AbsCorr = INTEGRATION

Data completeness= 0.998   Theta(max)= 25.999

R(reflections)= 0.0472(2998)   wR2(reflections)= 0.1224(3543)

S = 1.160   Npar= 280

The following ALERTS were generated. Each ALERT has the format
test-name_ALERT_alert-type_alert-level.
Click on the hyperlinks for more details of the test.

Alert level B

```
CRYSS02_ALERT_3_B  The value of _exptl_crystal_size_min is > 0.6
   Minimum crystal size given =   1.000
```

Author Response: For this and the other two related crystal size alerts: The compound was crystallized inside a glass capillary. The dimensions of the crystal itself were smaller, but the dimensions of the relevant capillary section are reported, because this volume of the capillary was filled with solid and liquid of the composition of the crystal and thus contributed to the absorption.

```
CRYSS02_ALERT_3_B  The value of _exptl_crystal_size_mid is > 0.8
   Mid crystal size given     =   1.000
```

Author Response: For this and the other two related crystal size alerts: The compound was crystallized inside a glass capillary. The dimensions of the crystal itself were smaller, but the dimensions of the relevant capillary section are reported, because this volume of the capillary was filled with solid and liquid of the composition of the crystal and thus contributed to the absorption.

```
CRYSS02_ALERT_3_B  The value of _exptl_crystal_size_max is > 1.0
   Maximum crystal size given =   5.000
```

Author Response: For this and the other two related crystal size alerts: The compound was crystallized inside a glass capillary. The dimensions of the crystal itself were smaller, but the dimensions of the relevant capillary section are reported, because this volume of the capillary was filled with solid and liquid of the composition of the crystal and thus contributed to the absorption.

Alert level C

```
PLAT420_ALERT_2_C D-H Bond Without Acceptor  N1       --H1N      .     Please Check
PLAT906_ALERT_3_C Large K Value in the Analysis of Variance ......      6.468 Check
PLAT911_ALERT_3_C Missing FCF Refl Between Thmin & STh/L=    0.600          8 Report
PLAT913_ALERT_3_C Missing # of Very Strong Reflections in FCF ....          4 Note
```

Alert level G

```
PLAT002_ALERT_2_G Number of Distance or Angle Restraints on AtSite         25 Note
PLAT063_ALERT_4_G Crystal Size Possibly too Large for Beam Size ..       5.00 mm
PLAT068_ALERT_1_G Reported F000 Differs from Calcd (or Missing)...     Please Check
PLAT171_ALERT_4_G The CIF-Embedded .res File Contains EADP Records          5 Report
PLAT174_ALERT_4_G The CIF-Embedded .res File Contains FLAT Records          2 Report
PLAT176_ALERT_4_G The CIF-Embedded .res File Contains SADI Records         11 Report
```

```
PLAT187_ALERT_4_G The CIF-Embedded .res File Contains RIGU Records          2 Report
PLAT190_ALERT_3_G A Non-default RIGU Restraint Value for First Par     0.0010 Report
PLAT190_ALERT_3_G A Non-default RIGU Restraint Value for First Par     0.0010 Report
PLAT191_ALERT_3_G A Non-default SADI Restraint Value has been used     0.0010 Report
PLAT191_ALERT_3_G A Non-default SADI Restraint Value has been used     0.0010 Report
PLAT191_ALERT_3_G A Non-default SADI Restraint Value has been used     0.0010 Report
PLAT191_ALERT_3_G A Non-default SADI Restraint Value has been used     0.0010 Report
PLAT191_ALERT_3_G A Non-default SADI Restraint Value has been used     0.0010 Report
PLAT191_ALERT_3_G A Non-default SADI Restraint Value has been used     0.0010 Report
PLAT191_ALERT_3_G A Non-default SADI Restraint Value has been used     0.0010 Report
PLAT191_ALERT_3_G A Non-default SADI Restraint Value has been used     0.0010 Report
PLAT191_ALERT_3_G A Non-default SADI Restraint Value has been used     0.0010 Report
PLAT191_ALERT_3_G A Non-default SADI Restraint Value has been used     0.0050 Report
PLAT191_ALERT_3_G A Non-default SADI Restraint Value has been used     0.0100 Report
PLAT301_ALERT_3_G Main Residue  Disorder .............(Resd  1  )        38% Note
PLAT302_ALERT_4_G Anion/Solvent/Minor-Residue Disorder (Resd  2  )      100% Note
PLAT302_ALERT_4_G Anion/Solvent/Minor-Residue Disorder (Resd  3  )      100% Note
PLAT302_ALERT_4_G Anion/Solvent/Minor-Residue Disorder (Resd  4  )      100% Note
PLAT304_ALERT_4_G Non-Integer Number of Atoms in ..... (Resd  2  )      2.61 Check
PLAT304_ALERT_4_G Non-Integer Number of Atoms in ..... (Resd  3  )      1.83 Check
PLAT304_ALERT_4_G Non-Integer Number of Atoms in ..... (Resd  4  )      0.56 Check
PLAT432_ALERT_2_G Short Inter X...Y Contact  O2       ..C11B     .      3.00 Ang.
                                                    x,y,z  =      1_555 Check
PLAT811_ALERT_5_G No ADDSYM Analysis: Too Many Excluded Atoms ....         ! Info
PLAT860_ALERT_3_G Number of Least-Squares Restraints ............        165 Note
PLAT899_ALERT_4_G SHELXL2018 is Deprecated and Succeeded by SHELXL     2019/3 Note
PLAT933_ALERT_2_G Number of HKL-OMIT Records in Embedded .res File          4 Note
PLAT961_ALERT_5_G Dataset Contains no Negative Intensities .......     Please Check
PLAT967_ALERT_5_G Note: Two-Theta Cutoff Value in Embedded .res ..       52.0 Degree
PLAT978_ALERT_2_G Number C-C Bonds with Positive Residual Density.          2 Info
```

---

0 **ALERT level A** = Most likely a serious problem - resolve or explain
3 **ALERT level B** = A potentially serious problem, consider carefully
4 **ALERT level C** = Check. Ensure it is not caused by an omission or oversight
35 **ALERT level G** = General information/check it is not something unexpected

1 ALERT type 1 CIF construction/syntax error, inconsistent or missing data
5 ALERT type 2 Indicator that the structure model may be wrong or deficient
21 ALERT type 3 Indicator that the structure quality may be low
12 ALERT type 4 Improvement, methodology, query or suggestion
3 ALERT type 5 Informative message, check

Datablock: as097b_01

Bond precision: C-C = 0.0047 A   Wavelength=0.71073

Cell:	a=10.1442(2)	b=15.5169(2)	c=15.9743(2)
	alpha=90	beta=90	gamma=90
Temperature:	180 K		

	Calculated	Reported
Volume	2514.46(7)	2514.46(7)
Space group	P 21 21 21	P 21 21 21
Hall group	P 2ac 2ab	P 2ac 2ab
Moiety formula	C14 H19 N3 O2 Si, 2(C H Cl3)	C14 H19 N3 O2 Si, 2(C H Cl3)
Sum formula	C16 H21 Cl6 N3 O2 Si	C16 H21 Cl6 N3 O2 Si
Mr	528.15	528.15
Dx,g cm-3	1.395	1.395
Z	4	4
Mu (mm-1)	0.748	0.748
F000	1080.0	1080.0
F000’	1084.14	
h,k,lmax	13,20,21	13,20,21
Nref	6076[3414]	6075
Tmin,Tmax	0.738,0.861	0.628,0.798
Tmin’	0.632	

Correction method= # Reported T Limits: Tmin=0.628 Tmax=0.798
AbsCorr = INTEGRATION

Data completeness= 1.78/1.00 Theta(max)= 27.999

R(reflections)= 0.0387(5459) wR2(reflections)= 0.0963(6075)

S = 1.097 Npar= 385

The following ALERTS were generated. Each ALERT has the format
test-name_ALERT_alert-type_alert-level.
Click on the hyperlinks for more details of the test.

Alert level C

```
PLAT241_ALERT_2_C High   'MainMol' Ueq as Compared to Neighbors of        C13 Check
PLAT242_ALERT_2_C Low    'MainMol' Ueq as Compared to Neighbors of        Si1 Check
PLAT242_ALERT_2_C Low    'MainMol' Ueq as Compared to Neighbors of         N3 Check
PLAT260_ALERT_2_C Large Average Ueq of Residue Including       Cl1      0.140 Check
PLAT260_ALERT_2_C Large Average Ueq of Residue Including      Cl1A      0.116 Check
PLAT260_ALERT_2_C Large Average Ueq of Residue Including      Cl1B      0.128 Check
PLAT340_ALERT_3_C Low Bond Precision on  C-C Bonds ...............    0.00467 Ang.
PLAT352_ALERT_3_C Short  N-H (X0.87,N1.01A)  N1       - H1N      .       0.76 Ang.
PLAT420_ALERT_2_C D-H Bond Without Acceptor  N1       --H1N      .     Please Check
PLAT911_ALERT_3_C Missing FCF Refl Between Thmin & STh/L=    0.600          3 Report
```

Alert level G

```
PLAT002_ALERT_2_G Number of Distance or Angle Restraints on AtSite         24 Note
PLAT153_ALERT_1_G The s.u.'s on the Cell Axes   are Equal ..(Note)     0.0002 Ang.
```

```
PLAT171_ALERT_4_G The CIF-Embedded .res File Contains EADP Records          2 Report
PLAT176_ALERT_4_G The CIF-Embedded .res File Contains SADI Records          4 Report
PLAT187_ALERT_4_G The CIF-Embedded .res File Contains RIGU Records          4 Report
PLAT190_ALERT_3_G A Non-default RIGU Restraint Value for First Par     0.0010 Report
PLAT190_ALERT_3_G A Non-default RIGU Restraint Value for First Par     0.0010 Report
PLAT190_ALERT_3_G A Non-default RIGU Restraint Value for First Par     0.0010 Report
PLAT190_ALERT_3_G A Non-default RIGU Restraint Value for First Par     0.0010 Report
PLAT191_ALERT_3_G A Non-default SADI Restraint Value has been used     0.0010 Report
PLAT191_ALERT_3_G A Non-default SADI Restraint Value has been used     0.0010 Report
PLAT191_ALERT_3_G A Non-default SADI Restraint Value has been used     0.0050 Report
PLAT191_ALERT_3_G A Non-default SADI Restraint Value has been used     0.0100 Report
PLAT302_ALERT_4_G Anion/Solvent/Minor-Residue Disorder (Resd  2  )       100% Note
PLAT302_ALERT_4_G Anion/Solvent/Minor-Residue Disorder (Resd  3  )       100% Note
PLAT302_ALERT_4_G Anion/Solvent/Minor-Residue Disorder (Resd  4  )       100% Note
PLAT302_ALERT_4_G Anion/Solvent/Minor-Residue Disorder (Resd  5  )       100% Note
PLAT302_ALERT_4_G Anion/Solvent/Minor-Residue Disorder (Resd  6  )       100% Note
PLAT302_ALERT_4_G Anion/Solvent/Minor-Residue Disorder (Resd  7  )       100% Note
PLAT304_ALERT_4_G Non-Integer Number of Atoms in ..... (Resd  2  )       2.65 Check
PLAT304_ALERT_4_G Non-Integer Number of Atoms in ..... (Resd  3  )       2.07 Check
PLAT304_ALERT_4_G Non-Integer Number of Atoms in ..... (Resd  4  )       1.19 Check
PLAT304_ALERT_4_G Non-Integer Number of Atoms in ..... (Resd  5  )       1.74 Check
PLAT304_ALERT_4_G Non-Integer Number of Atoms in ..... (Resd  6  )       1.32 Check
PLAT304_ALERT_4_G Non-Integer Number of Atoms in ..... (Resd  7  )       1.03 Check
PLAT432_ALERT_2_G Short Inter X...Y Contact  O2       ..C16A     .       2.97 Ang.
                                                      x,y,z  =      1_555 Check
PLAT811_ALERT_5_G No ADDSYM Analysis: Too Many Excluded Atoms ....          ! Info
PLAT860_ALERT_3_G Number of Least-Squares Restraints ............         206 Note
PLAT899_ALERT_4_G SHELXL2018 is Deprecated and Succeeded by SHELXL     2019/3 Note
PLAT913_ALERT_3_G Missing # of Very Strong Reflections in FCF ....          1 Note
PLAT961_ALERT_5_G Dataset Contains no Negative Intensities .......     Please Check
PLAT967_ALERT_5_G Note: Two-Theta Cutoff Value in Embedded .res ..       56.0 Degree
PLAT978_ALERT_2_G Number C-C Bonds with Positive Residual Density.          3 Info
PLAT992_ALERT_5_G Repd & Actual _reflns_number_gt Values Differ by          2 Check
```

```
   0 ALERT level A = Most likely a serious problem - resolve or explain
   0 ALERT level B = A potentially serious problem, consider carefully
  10 ALERT level C = Check. Ensure it is not caused by an omission or oversight
  34 ALERT level G = General information/check it is not something unexpected

   1 ALERT type 1 CIF construction/syntax error, inconsistent or missing data
  10 ALERT type 2 Indicator that the structure model may be wrong or deficient
  13 ALERT type 3 Indicator that the structure quality may be low
  16 ALERT type 4 Improvement, methodology, query or suggestion
   4 ALERT type 5 Informative message, check
```

---

checkCIF publication errors

Alert level A

```
PUBL004_ALERT_1_A  The contact author's name and address are missing,
            _publ_contact_author_name and _publ_contact_author_address.
PUBL005_ALERT_1_A  _publ_contact_author_email, _publ_contact_author_fax and
            _publ_contact_author_phone are all missing.
```

```
            At least one of these should be present.
PUBL006_ALERT_1_A  _publ_requested_journal is missing
            e.g. 'Acta Crystallographica Section C'
PUBL008_ALERT_1_A  _publ_section_title is missing. Title of paper.
PUBL009_ALERT_1_A  _publ_author_name is missing. List of author(s) name(s).
PUBL010_ALERT_1_A  _publ_author_address is missing. Author(s) address(es).
PUBL012_ALERT_1_A  _publ_section_abstract is missing.
            Abstract of paper in English.
```

Alert level G

```
PUBL017_ALERT_1_G The _publ_section_references section is missing or
            empty.
```

---

   7 **ALERT level A** = Data missing that is essential or data in wrong format
   1 **ALERT level G** = General alerts. Data that may be required is missing

---

Publication of your CIF

You should attempt to resolve as many as possible of the alerts in all categories. Often the minor alerts point to easily fixed oversights, errors and omissions in your CIF or refinement strategy, so attention to these fine details can be worthwhile. In order to resolve some of the more serious problems it may be necessary to carry out additional measurements or structure refinements. However, the nature of your study may justify the reported deviations from journal submission requirements and the more serious of these should be commented upon in the discussion or experimental section of a paper or in the "special_details" fields of the CIF. *checkCIF* was carefully designed to identify outliers and unusual parameters, but every test has its limitations and alerts that are not important in a particular case may appear. Conversely, the absence of alerts does not guarantee there are no aspects of the results needing attention. It is up to the individual to critically assess their own results and, if necessary, seek expert advice.

If level A alerts remain, which you believe to be justified deviations, and you intend to submit this CIF for publication in a journal, you should additionally insert an explanation in your CIF using the Validation Reply Form (VRF) below. This will allow your explanation to be considered as part of the review process.

Validation response form

Please find below a validation response form (VRF) that can be filled in and pasted into your CIF.

```
# start Validation Reply Form
_vrf_PUBL004_GLOBAL
;
PROBLEM: The contact author's name and address are missing,
RESPONSE: ...
;
_vrf_PUBL005_GLOBAL
;
PROBLEM: _publ_contact_author_email, _publ_contact_author_fax and
RESPONSE: ...
```

```
;
_vrf_PUBL006_GLOBAL
;
PROBLEM: _publ_requested_journal is missing
RESPONSE: ...
;
_vrf_PUBL008_GLOBAL
;
PROBLEM: _publ_section_title is missing. Title of paper.
RESPONSE: ...
;
_vrf_PUBL009_GLOBAL
;
PROBLEM: _publ_author_name is missing. List of author(s) name(s).
RESPONSE: ...
;
_vrf_PUBL010_GLOBAL
;
PROBLEM: _publ_author_address is missing. Author(s) address(es).
RESPONSE: ...
;
_vrf_PUBL012_GLOBAL
;
PROBLEM: _publ_section_abstract is missing.
RESPONSE: ...
;
# end Validation Reply Form
```

If you wish to submit your CIF for publication in Acta Crystallographica Section C or E, you should upload your CIF via the web. If you wish to submit your CIF for publication in IUCrData you should upload your CIF via the web. If your CIF is to form part of a submission to another IUCr journal, you will be asked, either during electronic submission or by the Co-editor handling your paper, to upload your CIF via our web site.

PLATON version of 06/07/2023; check.def file version of 30/06/2023

Datablock as102b_01 - ellipsoid plot

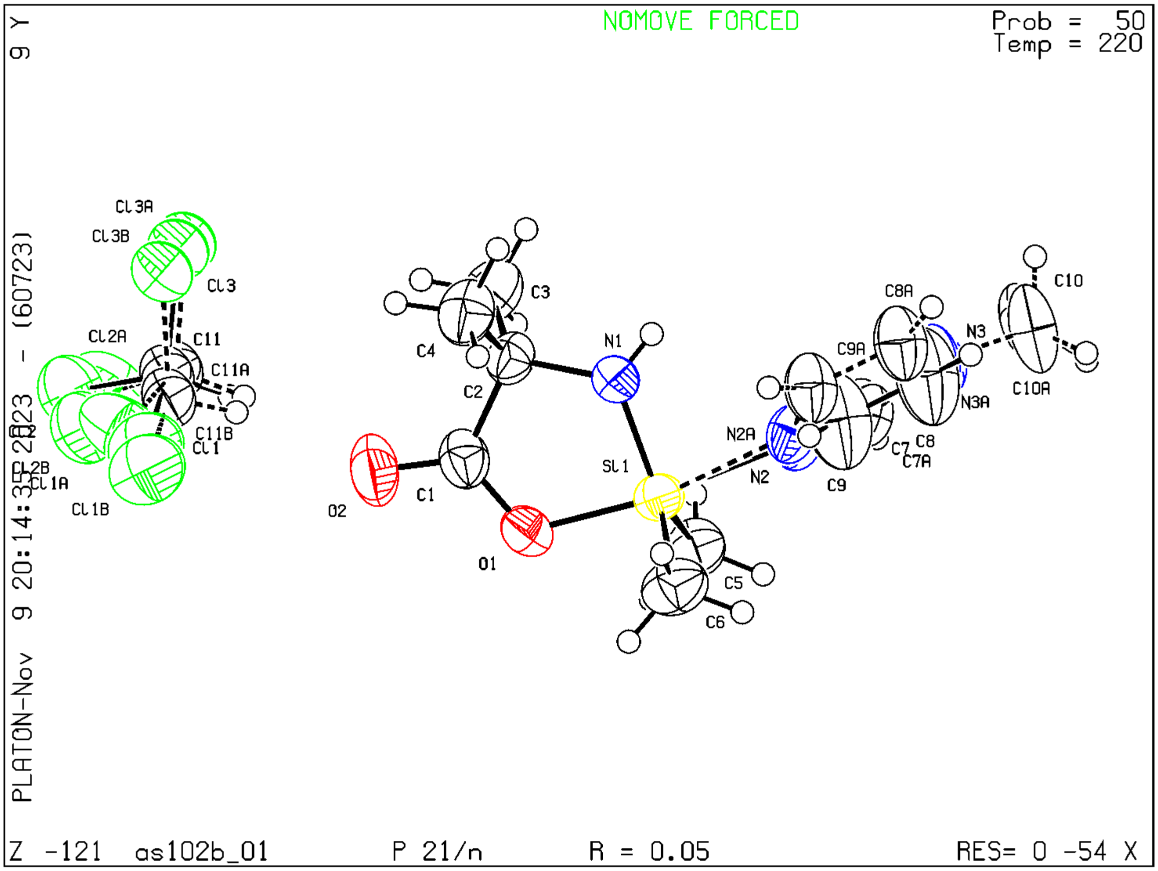

Datablock as097b_01 - ellipsoid plot

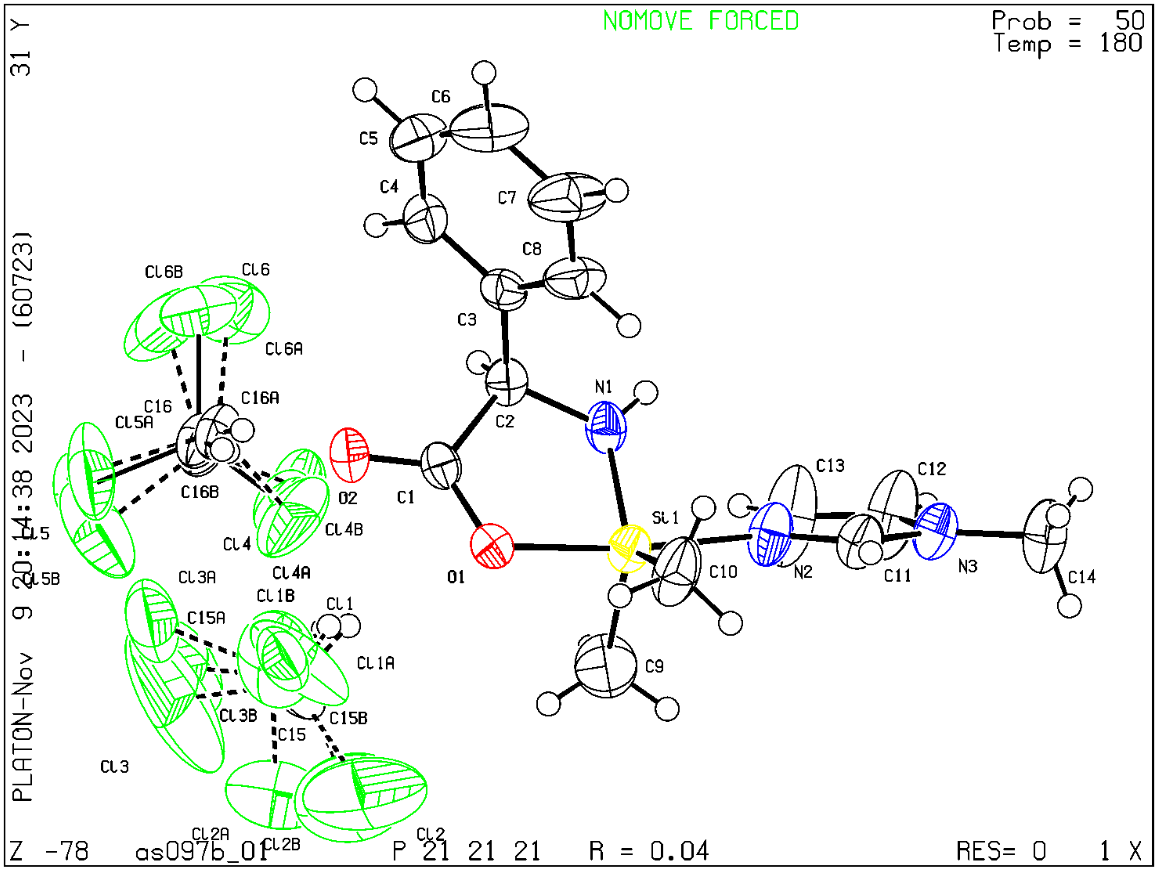

Supplement: Supplementary file 1 [file molecules-28-07816-s001.zip › checkcif_all-in-one.pdf]
