# Supplementary material for: Lewis Acid-Base Adducts of α-Amino Acid-Derived Silaheterocycles and N-Methylimidazole
Source: Molecules. 2023 Nov 28;28(23):7816. doi: 10.3390/molecules28237816 (PMC10708346; doi:10.3390/molecules28237816)
Supplement: Supplementary file 1 [file molecules-28-07816-s001.zip › Supporting_information_NMI-Adducts.pdf]

Lewis Acid-Base-Adducts of α-Amino Acid Derived Silaheterocycles and N-Methylimidazole

*Anne Seidel [1], Robert Gericke [2], Beate Kutzner [1] and Jörg Wagler [1,]**

[1] Technische Universität Bergakademie Freiberg, Institut für Anorganische Chemie, Leipziger Straße 29, 09596 Freiberg, Germany

[2] Helmholtz-Zentrum Dresden-Rossendorf e.V., Institute of Resource Ecology, Bautzner Landstraße 400, 01328 Dresden, Germany

Supporting Information:

Content:

- $^{29}Si\{^1H\}$ NMR spectra of $CDCl_3$ solutions of the isolated compounds **(Aib)SiMe$_2$-NMI · CHCl$_3$** and **(Phg)SiMe$_2$-NMI · 2CHCl$_3$**.
- ^1H $^{13}C\{^1H\}$ and $^{29}Si\{^1H\}$ INEPT NMR spectra of raw $CDCl_3$ solutions of the syntheses of **(Aib)SiMe$_2$-NMI**, **(Phg)SiMe$_2$-NMI** and **(Val)SiMe$_2$-NMI**.
- Graphics and tables with Cartesian coordinates of optimized molecular structures of compounds **(*Amac*)SiMe$_2$-NMI**, **(*Amac*)SiMe$_2$-CHCl$_3$**, **(*Amac*)SiMe$_2$-NMI-CHCl$_3$** and **(*Amac*)SiMe$_2$** (*Amac* = Aib, Phg, Val).

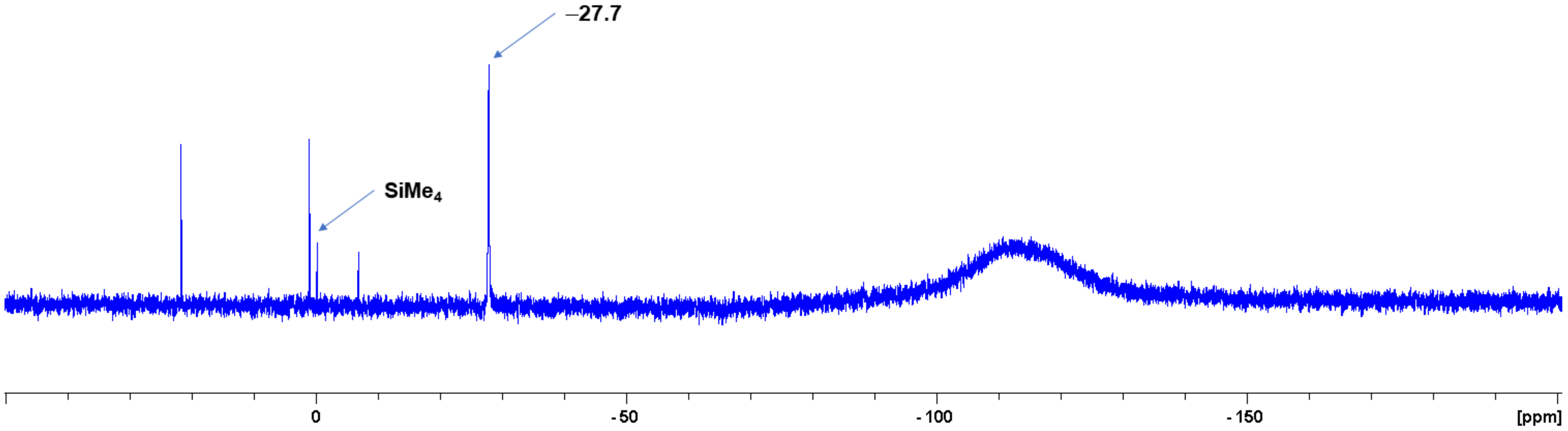

Figure S1. $^{29}Si\{^{1}H\}$ NMR spectrum of a solution of previously isolated **(Aib)SiMe$_2$-NMI · CHCl$_3$** in $CDCl_3$.

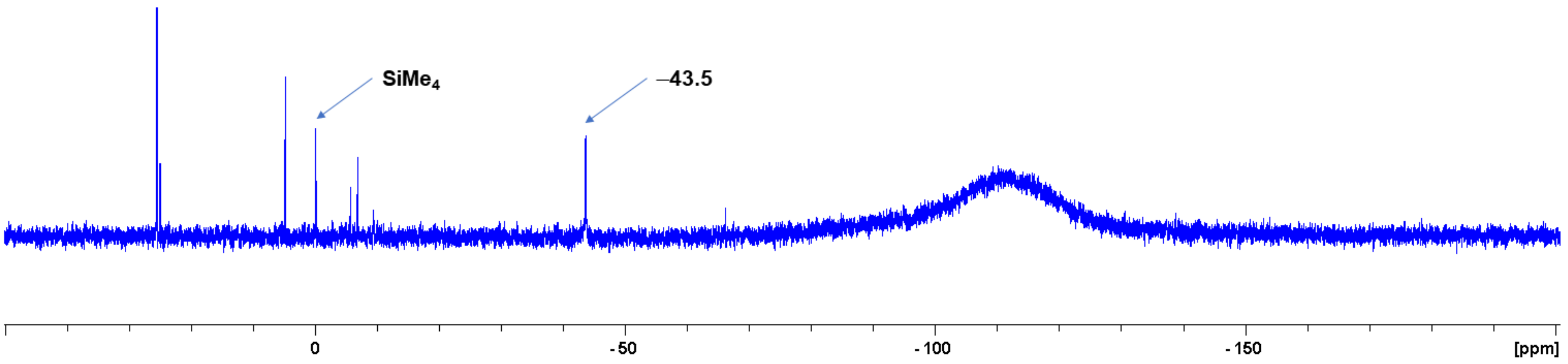

Figure S2. $^{29}Si\{^1H\}$ NMR spectrum of a solution of previously isolated **(Phg)SiMe$_2$-NMI · 2CHCl$_3$** in $CDCl_3$.

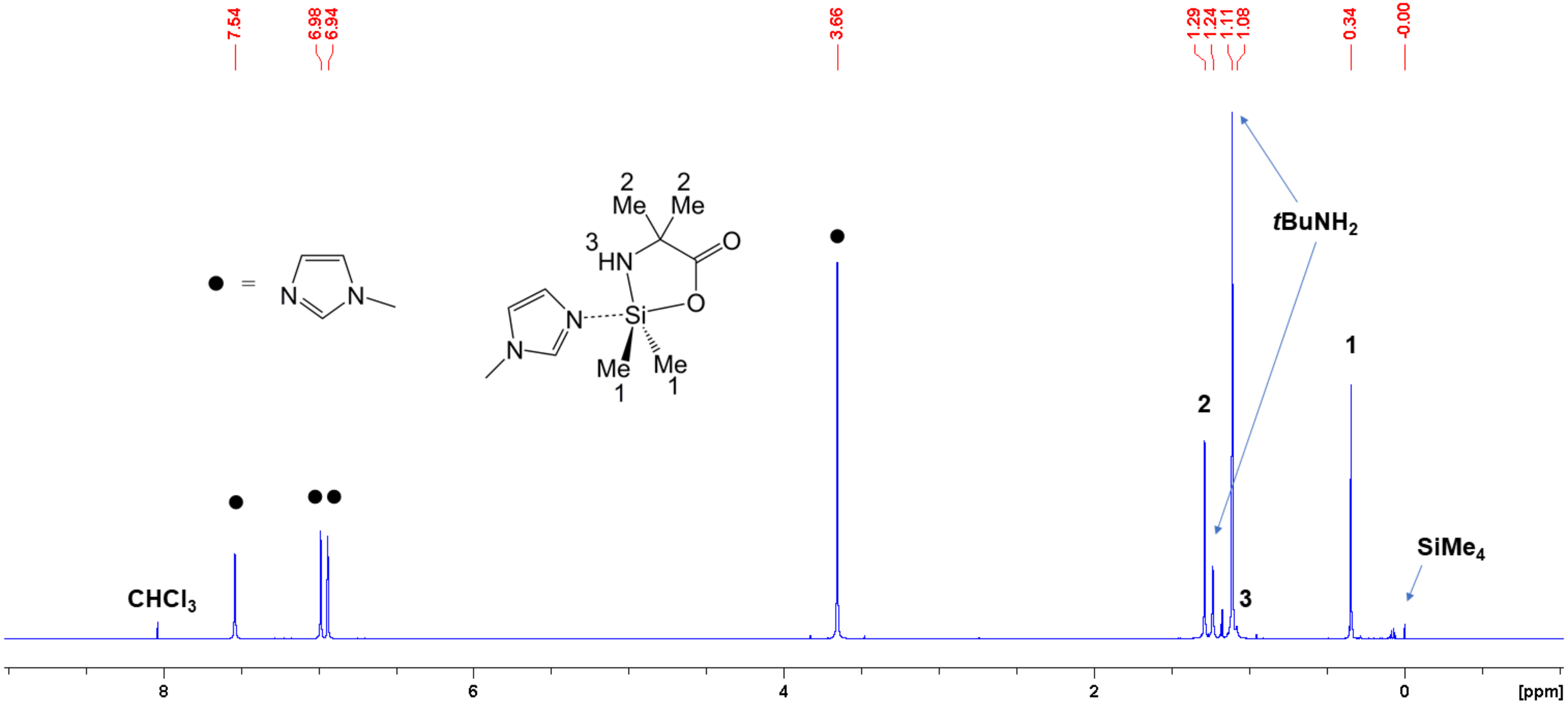

Figure S3. ^{1}H NMR spectrum of a solution of **(Aib)SiMe$_2$-NMI** in $CDCl_3$ (in the presence of excess NMI and the reaction product $tBuNH_2$). According to the amounts of starting materials used, the molar ratio of **(Aib)SiMe$_2$** : NMI : $tBuNH_2$ should be close to 1 : 4 : 2 (cf. sample **(Aib)SiMe$_2$-NMI-4** mentioned in the discussion).

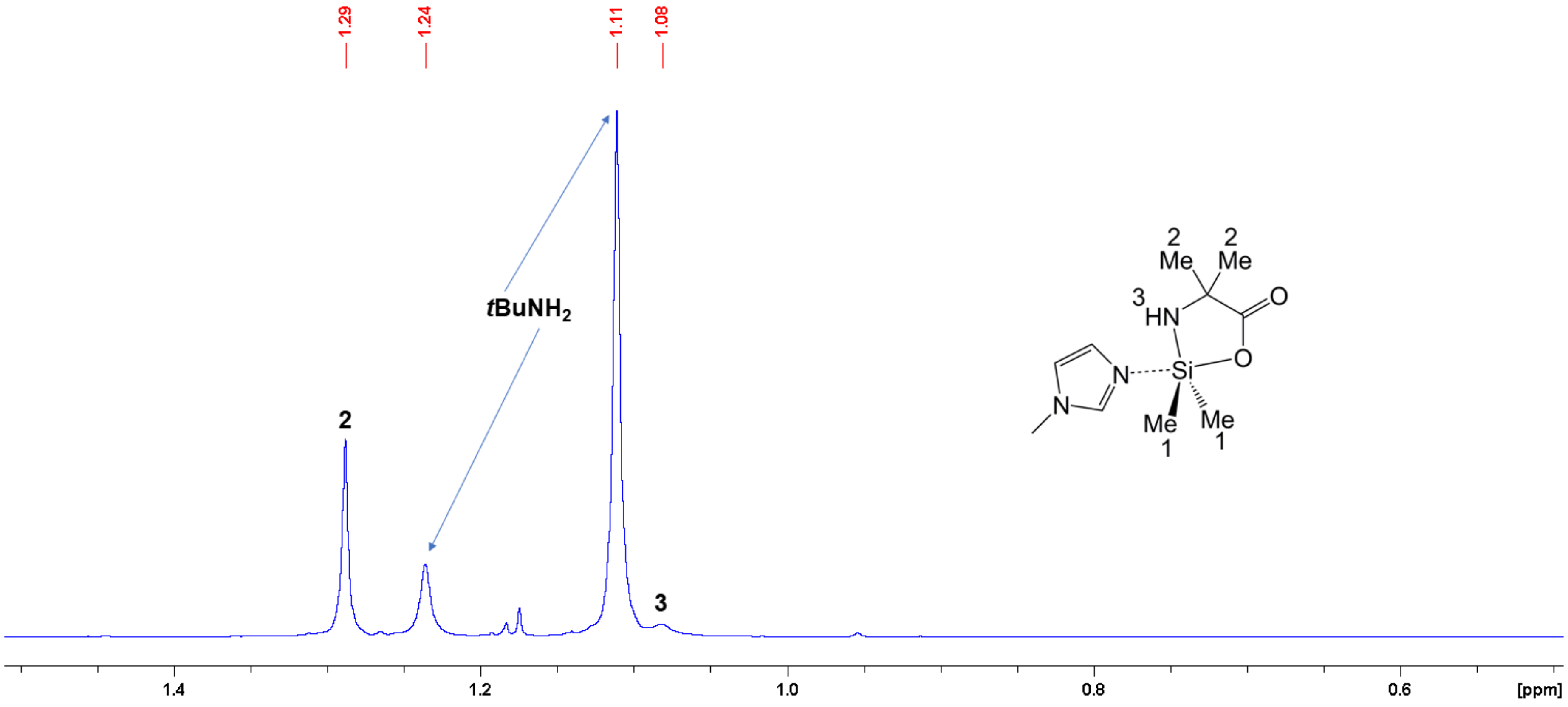

Figure S4. Magnified section of the ^{1}H NMR spectrum of a solution of **(Aib)SiMe$_2$-NMI** in $CDCl_3$ (in the presence of excess NMI and the reaction product $tBuNH_2$). According to the amounts of starting materials used, the molar ratio of **(Aib)SiMe$_2$** : NMI : $tBuNH_2$ should be close to 1 : 4 : 2 (cf. sample **(Aib)SiMe$_2$-NMI-4** mentioned in the discussion).

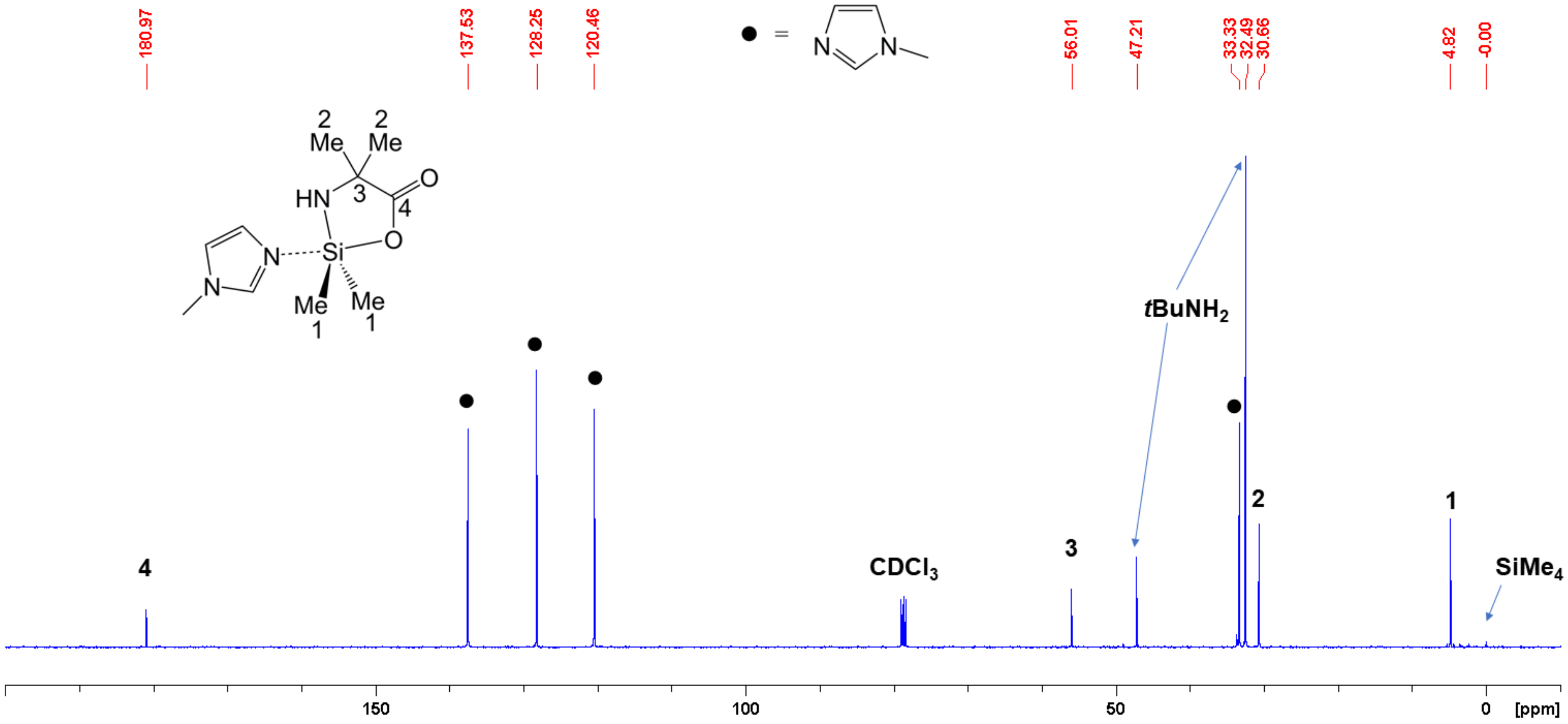

Figure S5. $^{13}C\{^{1}H\}$ NMR spectrum of a solution of **(Aib)SiMe2-NMI** in $CDCl_3$ (in the presence of excess NMI and the reaction product $tBuNH_2$). According to the amounts of starting materials used, the molar ratio of **(Aib)SiMe2** : NMI : $tBuNH_2$ should be close to 1 : 4 : 2 (cf. sample **(Aib)SiMe2-NMI-4** mentioned in the discussion).

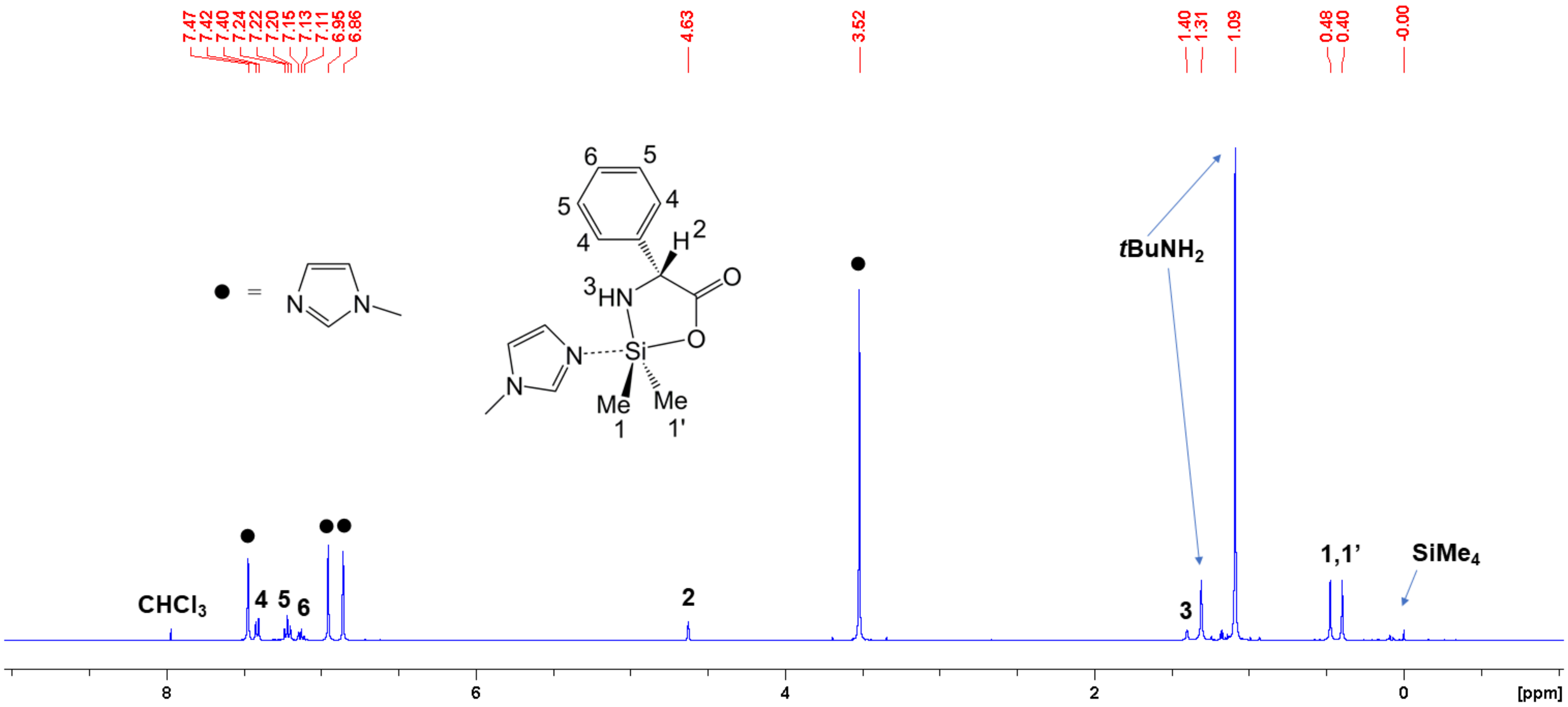

Figure S6. ^{1}H NMR spectrum of a solution of **(Phg)SiMe2-NMI** in $CDCl_3$ (in the presence of excess NMI and the reaction product $tBuNH_2$). According to the amounts of starting materials used, the molar ratio of **(Phg)SiMe2** : NMI : $tBuNH_2$ should be close to 1 : 4 : 2 (cf. sample **(Phg)SiMe2-NMI-4** mentioned in the discussion).

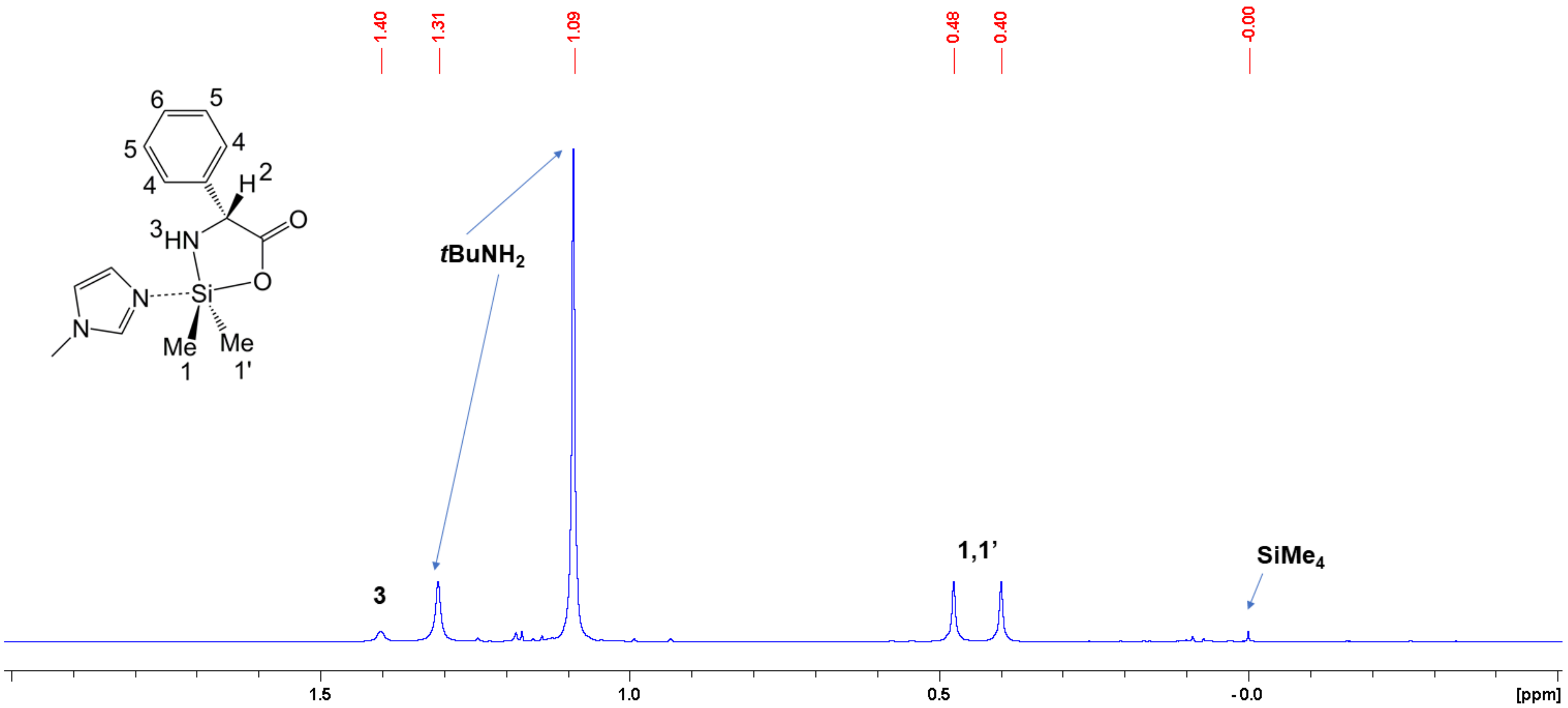

Figure 7. Magnified section of the ^{1}H NMR spectrum of a solution of **(Phg)SiMe$_2$-NMI** in $CDCl_3$ (in the presence of excess NMI and the reaction product tBuNH$_2$). According to the amounts of starting materials used, the molar ratio of **(Phg)SiMe$_2$** : NMI : tBuNH$_2$ should be close to 1 : 4 : 2 (cf. sample **(Phg)SiMe$_2$-NMI-4** mentioned in the discussion).

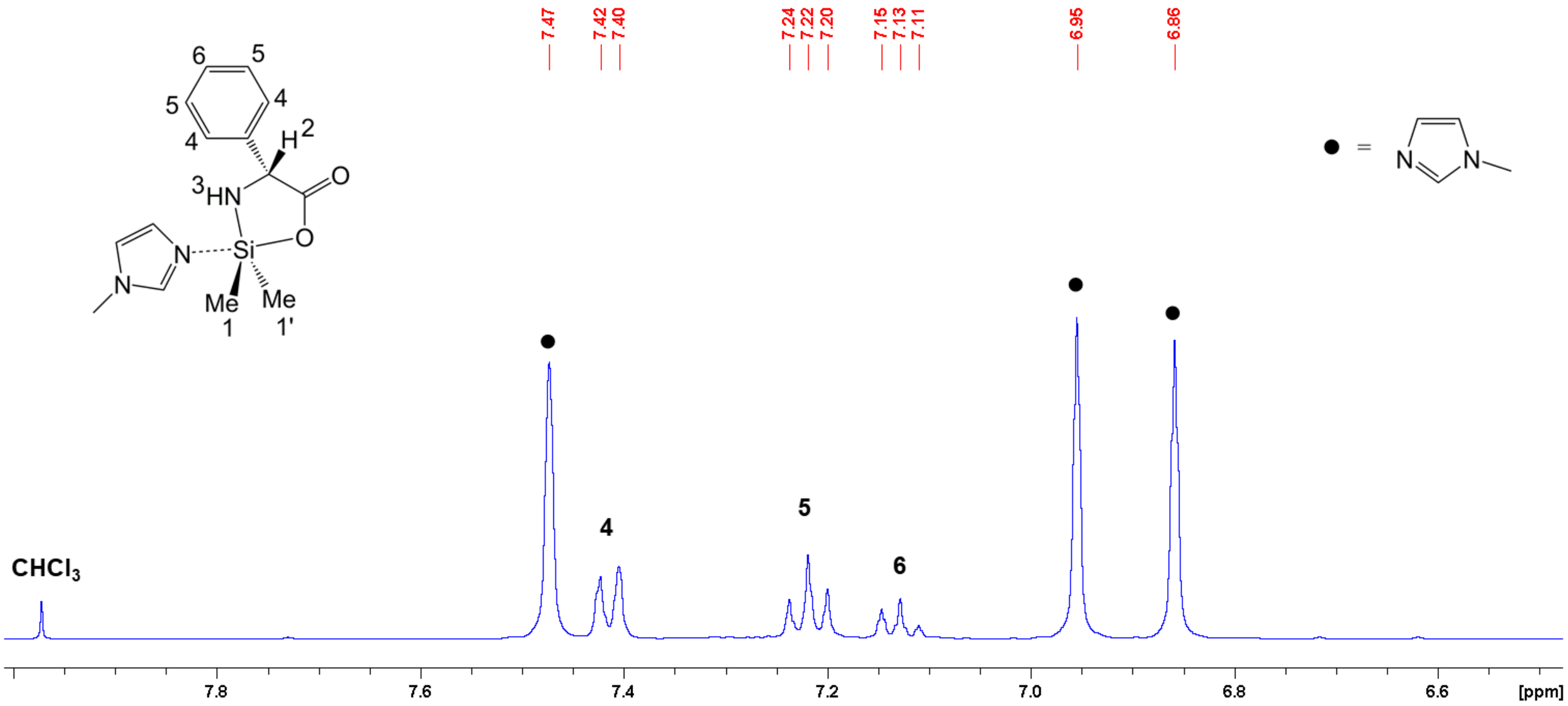

Figure S8. Magnified section of the ^{1}H NMR spectrum of a solution of **(Phg)SiMe$_2$-NMI** in $CDCl_3$ (in the presence of excess NMI and the reaction product $tBuNH_2$). According to the amounts of starting materials used, the molar ratio of **(Phg)SiMe$_2$** : NMI : $tBuNH_2$ should be close to 1 : 4 : 2 (cf. sample **(Phg)SiMe$_2$-NMI-4** mentioned in the discussion).

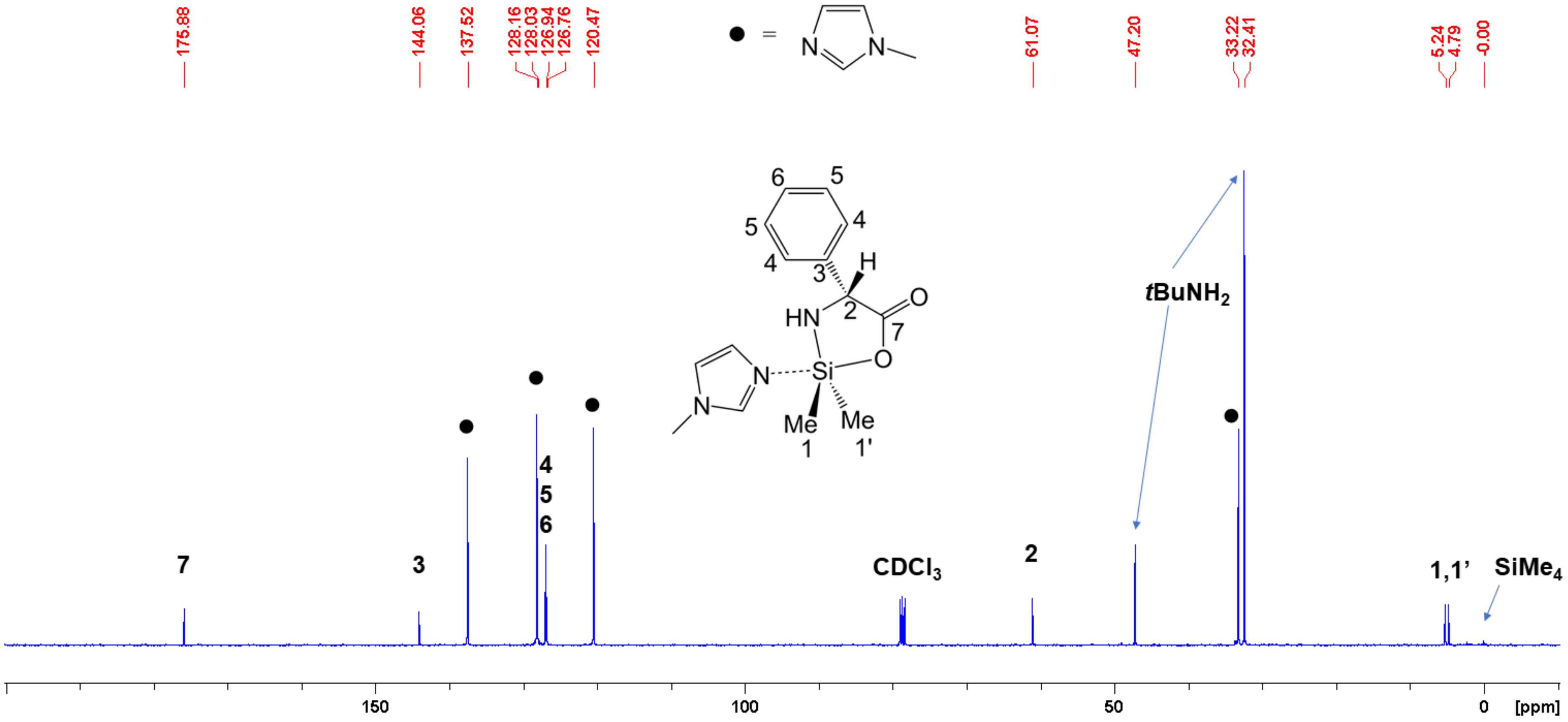

Figure S9. $^{13}C\{^1H\}$ NMR spectrum of a solution of **(Phg)SiMe$_2$-NMI** in $CDCl_3$ (in the presence of excess NMI and the reaction product $tBuNH_2$). According to the amounts of starting materials used, the molar ratio of **(Phg)SiMe$_2$** : NMI : $tBuNH_2$ should be close to 1 : 4 : 2 (cf. sample **(Phg)SiMe$_2$-NMI-4** mentioned in the discussion).

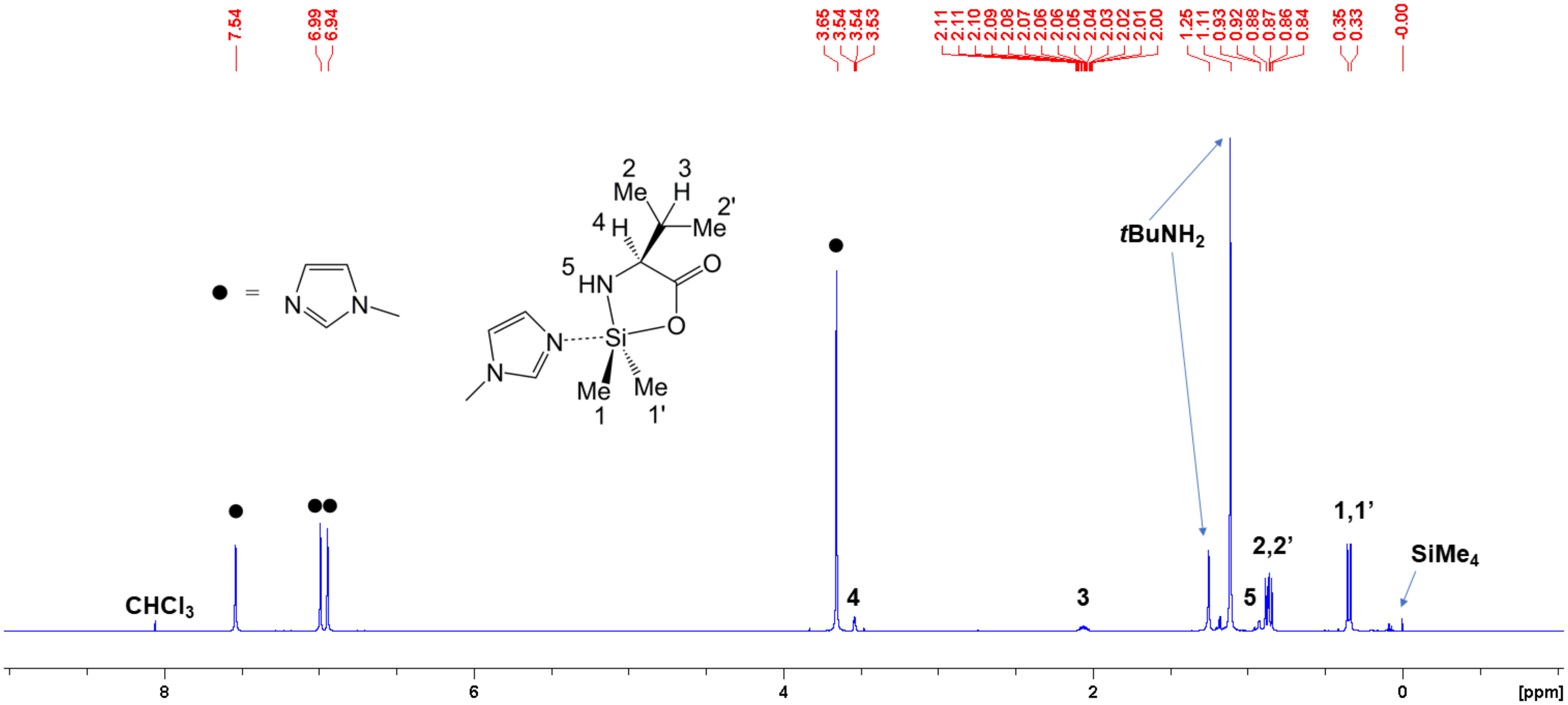

Figure S10. ^{1}H NMR spectrum of a solution of **(Val)SiMe$_2$-NMI** in $CDCl_3$ (in the presence of excess NMI and the reaction product $tBuNH_2$). According to the amounts of starting materials used, the molar ratio of **(Val)SiMe$_2$** : NMI : $tBuNH_2$ should be close to 1 : 4 : 2 (cf. sample **(Val)SiMe$_2$-NMI-4** mentioned in the discussion).

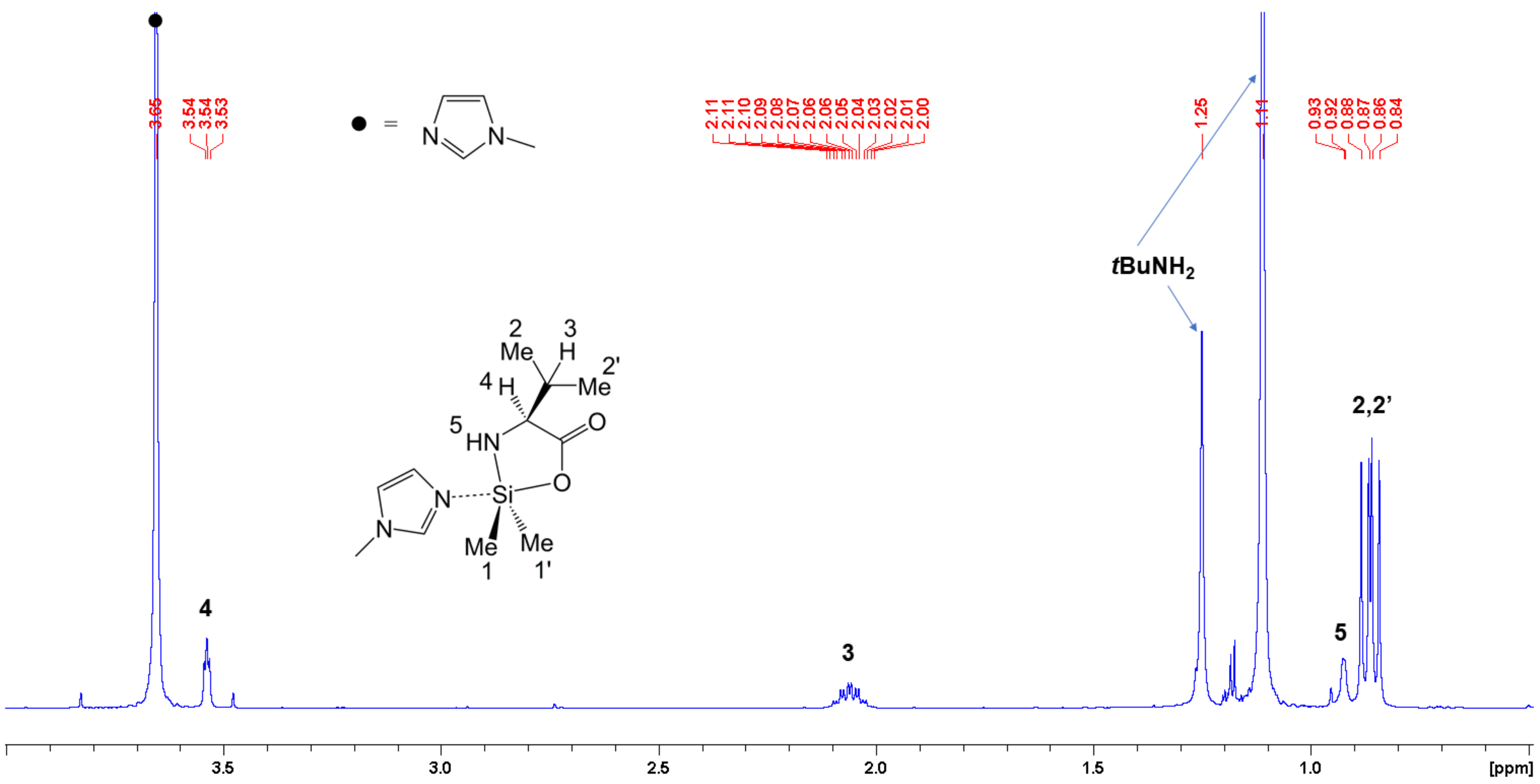

Figure 11. Magnified section of the ^{1}H NMR spectrum of a solution of **(Val)SiMe$_2$-NMI** in $CDCl_3$ (in the presence of excess NMI and the reaction product $tBuNH_2$). According to the amounts of starting materials used, the molar ratio of **(Val)SiMe$_2$** : NMI : $tBuNH_2$ should be close to 1 : 4 : 2 (cf. sample **(Val)SiMe$_2$-NMI-4** mentioned in the discussion).

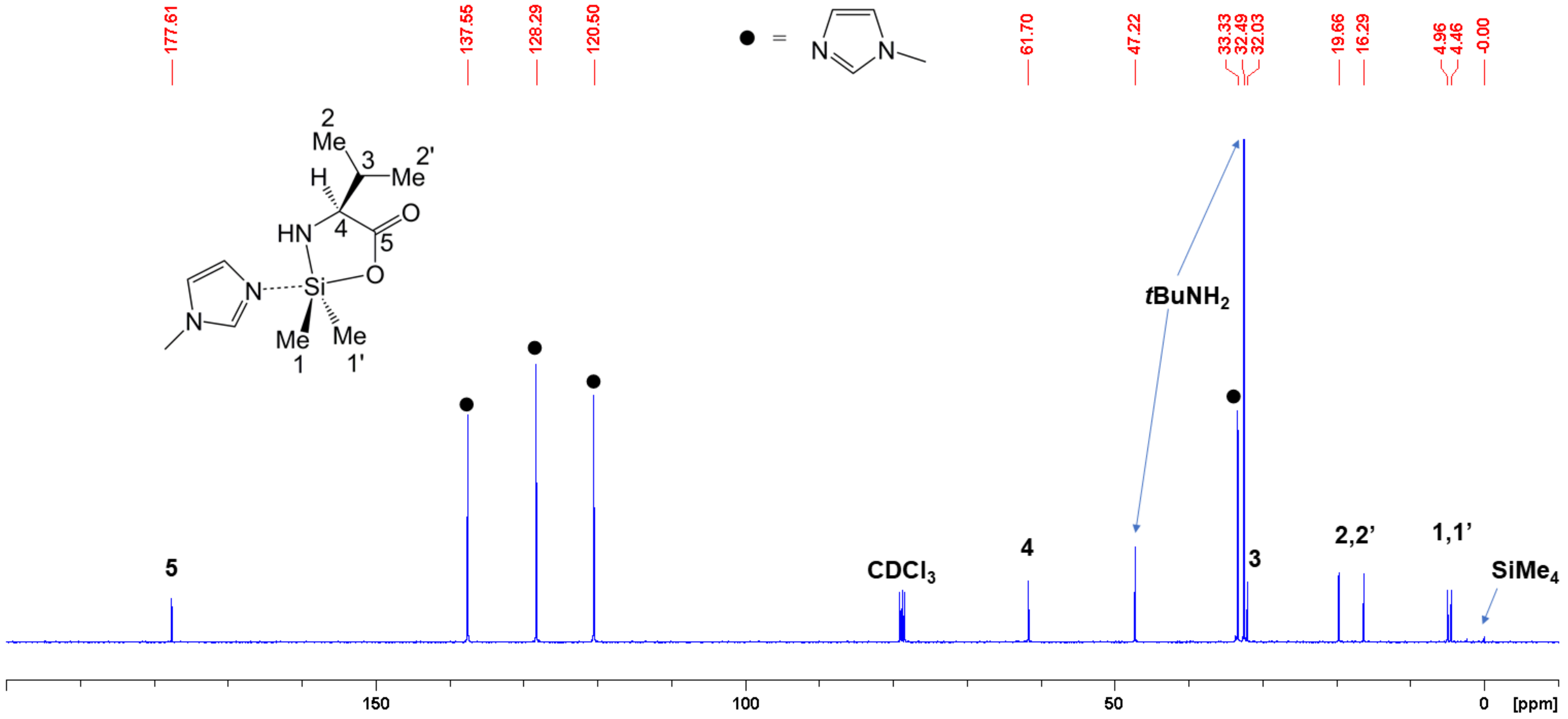

Figure S12. $^{13}C\{^1H\}$ NMR spectrum of a solution of **(Val)SiMe$_2$-NMI** in $CDCl_3$ (in the presence of excess NMI and the reaction product $tBuNH_2$). According to the amounts of starting materials used, the molar ratio of **(Val)SiMe$_2$** : NMI : $tBuNH_2$ should be close to 1 : 4 : 2 (cf. sample **(Val)SiMe$_2$-NMI-4** mentioned in the discussion).

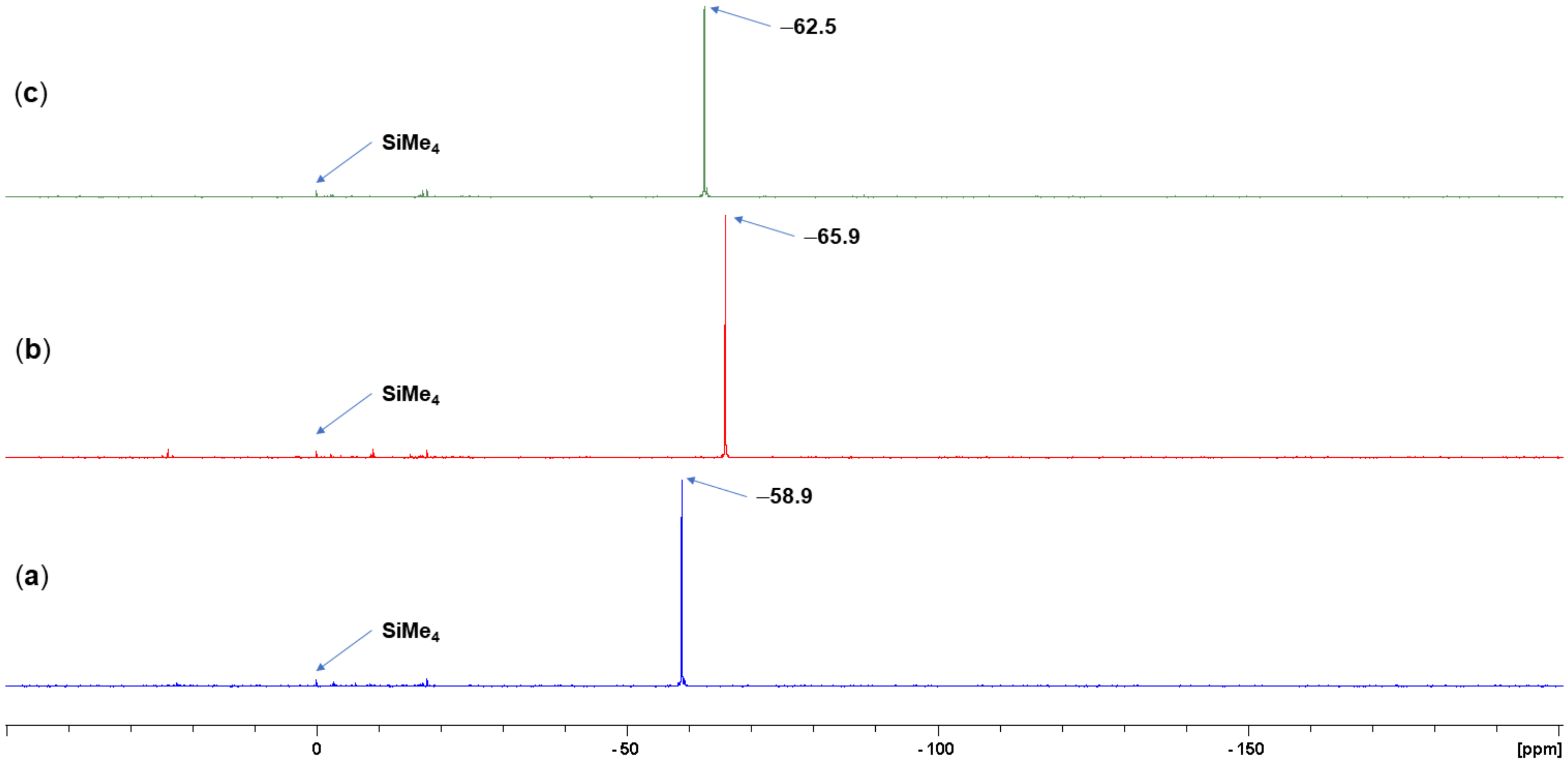

Figure S13. $^{29}Si\{^{1}H\}$ INEPT NMR spectra of solutions of (**a**) **(Val)SiMe$_2$-NMI**, (**b**) **(Phg)SiMe$_2$-NMI** and (**c**) **(Aib)SiMe$_2$-NMI** in $CDCl_3$, which were used for recording of the respective ^{1}H and $^{13}C\{^{1}H\}$ NMR spectra of Figures S3–S12.

Atomic coordinates and total energies:

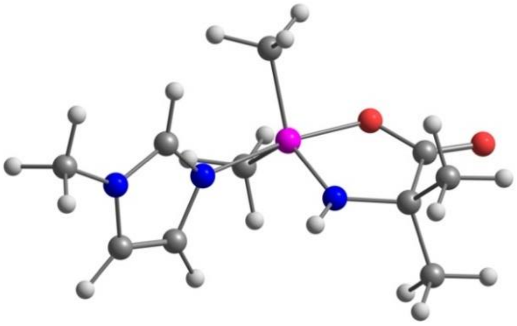

Figure S14.
Optimized molecular structure of **(Aib)SiMe$_2$-NMI**.

PBE0:
final single point energy: -997.773746057394 a.u.
final Gibbs free energy: -997.52771073 a.u.

Table S1. Atomic coordinates for optimized structure of **(Aib)SiMe$_2$-NMI**.

Si	-0.08088191809956	0.07611983219258	-0.00412147126033
O	-1.88461726478004	0.01238860392960	0.22687214978383
O	-3.49588705546657	-0.10557332177731	1.75445691257877
N	0.01708401180815	0.06524908436235	1.72069171060085
H	0.87121270368413	-0.00124507471485	2.24499597306460
N	1.99370419779679	0.19185620834174	0.06042991046465
N	4.12980315660641	-0.26517339697739	-0.03877800560774
C	-2.32022478509646	-0.04908846581599	1.45137326147073
C	-1.19790873834016	-0.04897807160289	2.49018552483254
C	-0.13326462735857	1.65400255999015	-1.02198550517108
H	-1.05528569687053	1.67984426838238	-1.60500774496786
H	0.71396297758845	1.74669191473894	-1.70253040866833
H	-0.13867622743797	2.52964819598012	-0.36684668624077
C	0.00446270120953	-1.48249691487577	-1.05147518056674
H	0.28038315791284	-2.35036948416683	-0.44609315070070
H	0.72180789571925	-1.39633900540532	-1.87004064320411
H	-0.97708026400422	-1.68271336432836	-1.48204584947587
C	2.88866034415428	-0.72855440914701	-0.24043813208067
H	2.67902750279461	-1.72282195124503	-0.59698197456575
C	4.02414710886850	1.02274540785710	0.41767832077172
H	4.89024111817639	1.61783394946308	0.65169263475270
C	2.69206752901922	1.29557127283705	0.47623331625122
H	2.19272444136276	2.19799686835312	0.78563794097747
C	5.35842694825563	-0.98904861821900	-0.28158454484501
H	5.11320770920519	-2.00681004544607	-0.57538959347323
H	5.96104404567006	-1.01345619842932	0.62520495083600
H	5.92421174627695	-0.51102109073237	-1.08053600754287
C	-1.25621371536423	-1.35998655013243	3.27386648041206
H	-2.20264766350856	-1.45353145664536	3.80763201562515
H	-1.14586829959785	-2.21305739711112	2.60170326709946
H	-0.44170230806822	-1.38672577067226	4.00219166594491
C	-1.39064385822313	1.13592943863019	3.43461852271679
H	-2.33972697512086	1.05874631249559	3.96616547321967
H	-0.58032773089933	1.15422764363623	4.16766415629085
H	-1.37354510187290	2.07516562425448	2.87902835067707

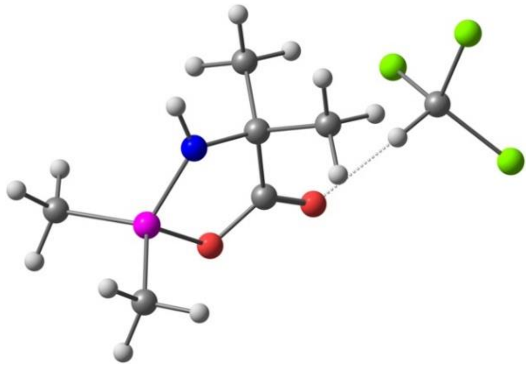

Figure S15.
Optimized molecular structure of **(Aib)SiMe$_2$-CHCl$_3$**.

PBE0:
final single point energy: -2157.895377070763 a.u.
final Gibbs free energy: -2157.73245359 a.u.

Table S2. Atomic coordinates for optimized structure of **(Aib)SiMe$_2$-CHCl$_3$**.

Si	0.67402391925024	1.43246868739297	0.41912531676802
Cl	-4.70683790199780	-0.72526863672304	-3.69321493946178
Cl	-4.74844736566849	-2.75711257449864	-1.62535794399668
Cl	-6.54937506332605	-0.48970820104895	-1.46178132228927
N	0.39210287803481	-0.25072008789436	0.31941927077832
O	-2.81615174899915	0.83737997809299	-0.68501970824328
O	-0.92070880847211	1.80339544668763	-0.06088615691626
C	-4.95998377165307	-1.04188390056140	-1.97842932102213
C	-0.81157688708383	-1.21831425324555	-1.61093901677298
C	1.86550842830787	2.14855109666914	-0.80402814141209
C	-1.66355582117534	0.73792839082163	-0.34824099616140
C	-0.91415014960428	-0.59285218132844	-0.21937678263812
C	-1.68948845904156	-1.51120577604372	0.72152456708893
C	0.95200838613595	2.10563037671211	2.11883688152634
H	-0.27936612883265	-0.55684124805636	-2.29649744690101
H	1.61898851519394	1.82908201737830	-1.81809061365127
H	-1.79977984670640	-1.42961709379825	-2.02002115010474
H	-4.20935583982744	-0.48326704741795	-1.42468659691207
H	-0.26093342156527	-2.15869055564757	-1.53982505286372
H	1.85760497965506	3.24033790199781	-0.76698233925532
H	2.88202128334758	1.81565758925411	-0.57748434870350
H	1.07536069190403	-0.98780011193998	0.30692888960348
H	-2.68221400760235	-1.72857253828073	0.32720194192754
H	-1.14685888409246	-2.45239356770014	0.82927681733518
H	-1.79018352610151	-1.05560084478061	1.70744711404311
H	0.92130827890206	3.19769913353179	2.11485412267980
H	1.93314820868069	1.80109444387195	2.49247498670855
H	0.19164620333757	1.73402285355525	2.80775760784636

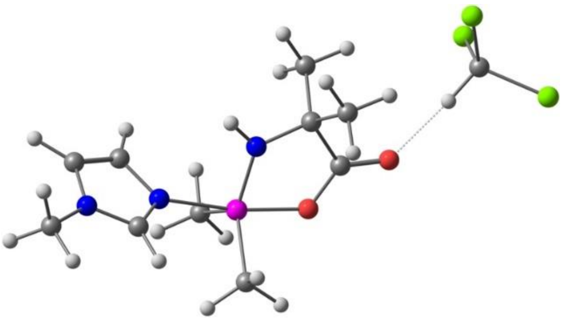

Figure S16.
Optimized molecular structure of **(Aib)SiMe$_2$-NMI-CHCl$_3$**.

PBE0:
final single point energy: -2423.450778006334 a.u.
final Gibbs free energy: -2423.19221366 a.u.

Table S3. Atomic coordinates for optimized structure of **(Aib)SiMe$_2$-NMI-CHCl$_3$**.

Si	0.98353944299011	1.19488550535240	0.49469278610292
Cl	-4.66661822522691	-0.46037734869722	-3.74680639668243
Cl	-4.75168154715033	-2.46486492680743	-1.65418545082002
Cl	-6.49108465650412	-0.15189529533525	-1.50930114805263
N	0.47728646264579	-0.44669200972436	0.30966159342387
N	2.81620966965163	0.40313022448060	1.00896056709474
N	4.56214130781765	-0.29108263407342	2.12523375438028
O	-2.67374873697385	0.79461846533642	-0.64141509906258
O	-0.71795196869149	1.62905068755425	-0.01352374187577
C	-4.91549252498172	-0.74806146280779	-2.02543703187901
C	-0.81253448376548	-1.36722731160265	-1.57639721286078
C	1.77524526317667	2.18445789810807	-0.89168294756807
C	-1.51059229418812	0.64733169293782	-0.30379580574353
C	-0.84781884829862	-0.72405250599404	-0.18863068206690
C	3.67989709618377	-0.19700451745199	0.12971883879638
C	4.77234379997272	-0.63236139525645	0.81437182941948
C	-1.65058702153141	-1.59435811126377	0.77534355255734
C	0.88812596262793	2.12452907561871	2.12424269595953
C	3.37678751230727	0.32723903889194	2.20030807463578
C	5.45959145667461	-0.54474882649821	3.23114584807495
H	-0.25950471715748	-0.73796393470538	-2.27608028744757
H	1.79831684901284	1.59561187406501	-1.81311715126486
H	-1.81931054657366	-1.52408461658214	-1.96498187304749
H	-4.14598144363373	-0.20216452712120	-1.48026364950851
H	-0.31271450687727	-2.33696259077645	-1.51514025200901
H	1.17243694792603	3.07208555781820	-1.09203380897880
H	3.45771592127492	-0.26948989474688	-0.92147704618608
H	2.79454635561689	2.49987616394838	-0.66559307619402
H	1.06395058215589	-1.24868657719583	0.45328415066135
H	5.66543432199285	-1.14124154641139	0.49427278618471
H	-2.66272441832403	-1.75786357353484	0.40452987001927
H	-1.16145766294492	-2.56553677421094	0.88301414702492
H	-1.70612667415181	-1.12575459805097	1.75939389610994
H	0.08332056322114	2.85868744207447	2.08066209957174
H	1.81607422557635	2.65220033311170	2.35395312232757
H	0.66784176340181	1.44661076701503	2.95359334101968
H	2.96151381342024	0.70113496381703	3.12074907550446
H	5.61452778070860	-1.61643589012983	3.34872832919084
H	6.41701497260444	-0.05659844903662	3.05365961149413
H	5.01649820601473	-0.14436037211502	4.13995269169414

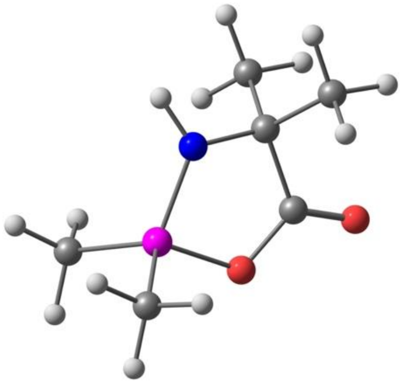

Figure S17.
Optimized molecular structure of **(Aib)SiMe$_2$**.

PBE0:
final single point energy: -732.219929966298 a.u.
final Gibbs free energy: -732.06836169 a.u.

Table S4. Atomic coordinates for optimized structure of **(Aib)SiMe$_2$**.

Si	-0.52760721198535	0.10188343335765	-0.07288414118817
O	-2.19324960117603	0.05077650850655	0.28097379041564
O	-3.59182203581275	-0.03219007753494	2.00799763745062
N	-0.09573390454233	0.22655242932827	1.57899518574736
H	0.81327076861360	0.05982047977062	1.97565003380268
C	-2.46714215924642	0.00984532439926	1.58848800794313
C	-1.21744376179630	-0.00027087655824	2.47738690008890
C	-0.16033010815431	1.59230906195023	-1.10471475799913
H	-0.61524483350519	1.50473102178148	-2.09401085700435
H	0.91927676998513	1.69541697302082	-1.24352622815252
H	-0.53207386814557	2.49818950347393	-0.62327452887332
C	-0.03853647373421	-1.45917445405629	-0.94340929421673
H	-0.36105303309540	-2.33537989508837	-0.37809701352165
H	1.04895804849952	-1.50253925721556	-1.04950097391290
H	-0.47220089139349	-1.50707228631919	-1.94487562666388
C	-1.14250574445484	-1.36794500978644	3.15892033644616
H	-2.01230386166623	-1.53022177366452	3.79628355925328
H	-1.09274311199359	-2.16951976380484	2.41971935542999
H	-0.24355315163388	-1.41198919004326	3.77761419559298
C	-1.33890433100993	1.10493690999584	3.52091988226694
H	-2.22320337296390	0.95328667852647	4.14018632056514
H	-0.45564548082371	1.09498177999128	4.16282618154244
H	-1.40579764996481	2.08148347996924	3.03963003498736

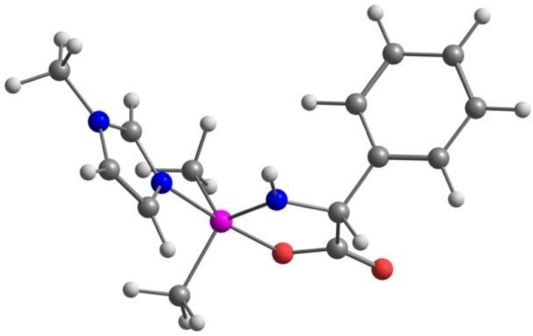

Figure S18.
Optimized molecular structure of **(Phg)SiMe$_2$-NMI**.

PBE0:
final single point energy: -1150.180944442688 a.u.
final Gibbs free energy: -1149.91101818 a.u.

Table S5. Atomic coordinates for optimized structure of **(Phg)SiMe$_2$-NMI**.

Si	-0.11780309862611	0.08530725205908	-0.08541467643146
O	-1.93342766521586	0.03720037800469	0.08883585926668
O	-3.59619094703078	0.02622151422284	1.56992528807897
N	-0.07937774216563	0.04616623395979	1.64560693822524
H	0.75616563091277	-0.02980069564907	2.19679007591769
N	1.94235215951655	0.17794288691295	0.05319036205647
N	4.07373107917648	-0.30744165671048	0.04776156953300
C	-2.41487367855310	0.00704653768553	1.29500511227795
C	-1.31630001063343	-0.05179108707142	2.36211257068928
H	-1.49196437244629	0.80940214485651	3.02000115866481
C	-1.47199885693237	-1.30348525431390	3.19743202352353
C	-2.13660792802807	-1.25340727766549	4.41723089131874
H	-2.51779289018274	-0.30332222232067	4.77498293246527
C	-2.31414604895977	-2.40231032458517	5.17482211248179
H	-2.83180567562991	-2.34622685090620	6.12521629299773
C	-1.82710859328844	-3.61881456906773	4.71827034470371
H	-1.96312021020806	-4.51698168756826	5.30875856529084
C	-1.16121656835888	-3.67650654859363	3.50091126537598
H	-0.77924101752148	-4.62307171425105	3.13645705515509
C	-0.98429965940164	-2.52594734466838	2.74743025269313
H	-0.46040795425220	-2.57043348507986	1.80005923439633
C	-0.13070301837810	1.68230772493727	-1.07270540340651
H	-1.02874459733476	1.71898418660340	-1.69129247076648
H	0.74266527488544	1.78696626705951	-1.71754498241065
H	-0.16290363170527	2.54625475772541	-0.40307149922970
C	-0.01505918159599	-1.45521376517561	-1.15631720182972
H	0.27198285842405	-2.33311822450395	-0.57099148025485
H	0.70598898490653	-1.34098291822310	-1.96847508919067
H	-0.99121568747454	-1.66066609822298	-1.59592930620504
C	2.83761766403419	-0.75385303135587	-0.21146885443068
H	2.63120631763494	-1.74350139560303	-0.58227968847848
C	3.96486804969485	0.97973103358995	0.50569247915942
H	4.82744526080997	1.56145564064126	0.78225307659949
C	2.63573690689396	1.27068852432332	0.50567777420703
H	2.13520558813490	2.17865550698960	0.79608561912645
C	5.30269707695436	-1.04858095105909	-0.13458896825709
H	5.05989635737049	-2.04923052990700	-0.48373814002072
H	5.83885652335881	-1.11889964982495	0.81080264572004
H	5.93204351321511	-0.55328415224418	-0.87293015401269

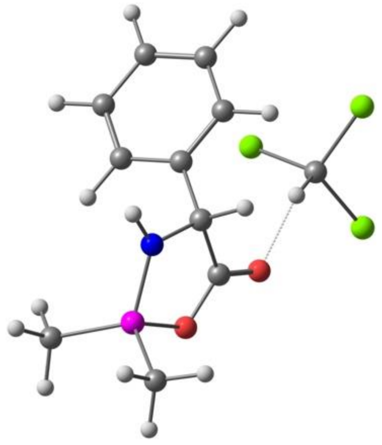

Figure S19.
Optimized molecular structure of **(Phg)SiMe$_2$-CHCl$_3$**.

PBE0:
final single point energy: -2310.302242593934 a.u.
final Gibbs free energy: -2310.11540654 a.u.

Table S6. Atomic coordinates for optimized structure of **(Phg)SiMe$_2$-CHCl$_3$**.

Si	-0.06153048826142	0.00430074443648	0.90079875169606
O	-1.74059123778789	0.10911786699109	0.60640992264242
O	-3.68810054924268	0.23380430378844	1.66870796012612
N	-0.29271144343930	-0.05831043205226	2.59424568447582
H	0.38569837291957	-0.18019126060188	3.32422761087725
C	-2.49104530562024	0.12734003062188	1.70447378873794
C	-1.67469854835704	0.01359979492736	2.99725279940526
H	-1.89889475568148	0.92990959419399	3.55899839222151
C	-2.15416565065070	-1.15863126367144	3.82446980329732
C	-2.96040716980403	-0.94321745571281	4.93422452725196
H	-3.23627498465587	0.07012409537979	5.20469105465749
C	-3.40937925314374	-2.01245494575060	5.69685809870731
H	-4.03957671971907	-1.83125802444174	6.55905030788691
C	-3.05385119494279	-3.30726484633578	5.35292263526291
H	-3.40247234685458	-4.14338922171033	5.94703374356691
C	-2.25042088382906	-3.52907355756943	4.24165990670599
H	-1.97250472025246	-4.53949885180459	3.96602895539919
C	-1.80173326625647	-2.46007126904275	3.48264791346683
H	-1.16322583335545	-2.63364265440066	2.62429945002330
C	0.76994531149520	1.52391218149950	0.25437164537842
H	0.71844615612025	1.56497478449529	-0.83604994576241
H	1.82611046297974	1.52006075932242	0.53745987223234
H	0.30902766134052	2.42443401213570	0.66343478288512
C	0.61108027700430	-1.52278179618629	0.10426035137288
H	0.04297644193039	-2.40607412858274	0.40017792238937
H	1.65310397286513	-1.66963524109619	0.40131116989158
H	0.58520544307982	-1.43720660883641	-0.98446052205509
C	-6.23235273177041	-0.75257306747403	3.21004144255887
Cl	-6.17409870490739	-2.51095254699368	3.16061211678655
Cl	-6.63866804347384	-0.18169699080148	4.82838829970487
Cl	-7.38213886353841	-0.13153126579464	2.02865722710390
H	-5.24937184019052	-0.37399395993219	2.94562660010503

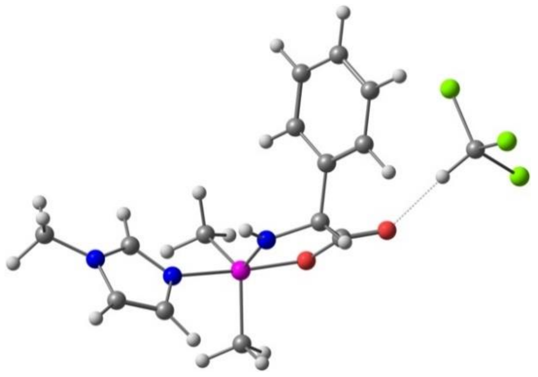

Figure S20.
Optimized molecular structure of **(Phg)SiMe$_2$-NMI-CHCl$_3$**.

PBE0:
final single point energy: -2575.859356730710 a.u.
final Gibbs free energy: -2575.57620347 a.u.

Table S7. Atomic coordinates for optimized structure of **(Phg)SiMe$_2$-NMI-CHCl$_3$**.

Si	0.27019762717249	-0.01252411353404	1.14626598947882
O	-1.51876566765400	0.08437606029104	0.74830085123799
O	-3.55229488700673	0.22352698003043	1.63359810954884
N	-0.24161833855949	-0.01895976368684	2.80012255187193
H	0.37063666293796	-0.13131620075968	3.58733383888552
N	2.17062906416346	-0.10312568093976	1.92327210778348
N	4.07550076019966	-0.83790555392475	2.70233472394201
C	-2.34399812858076	0.11901990054611	1.74361837408358
C	-1.64312308612882	0.02682772753845	3.09891304107208
H	-1.92750026063116	0.93660332905942	3.64489531029288
C	-2.15119850297110	-1.15632222888113	3.89173589710068
C	-2.96552607834098	-0.96518457272663	5.00001649707521
H	-3.23755264434601	0.04327798933250	5.29182167987423
C	-3.43040063157852	-2.04918083745185	5.73309053743761
H	-4.06663417731460	-1.88369800099189	6.59437732213733
C	-3.08453644416753	-3.33820374952150	5.36028551855513
H	-3.44653514460171	-4.18594620594313	5.92969245847033
C	-2.27555697835903	-3.53835272077170	4.24796554280280
H	-2.00867997080832	-4.54446362469778	3.94595545608476
C	-1.81080269552470	-2.45437567928576	3.52123150721442
H	-1.17289460214964	-2.61085793107649	2.65882800091951
C	0.69055581737529	1.56110548906052	0.21517811782027
H	0.02770703649614	1.66132621868136	-0.64561325338404
H	1.72335720471756	1.57792750815396	-0.13554579536426
H	0.53376170778233	2.43835241082556	0.84904831813967
C	0.55415636624916	-1.57176913100163	0.14016166066205
H	0.47248922240517	-2.46791562166696	0.76172073363974
H	1.53448618985273	-1.58084316977920	-0.34000652075157
H	-0.20611801325707	-1.64647414848240	-0.63799804884786
C	2.95145960437954	-1.15904057389131	2.04985306234799
H	2.73657146897348	-2.15195462263866	1.69221708113156
C	4.01185843532725	0.49563287845230	3.01275609905199
H	4.80601553868057	0.99646623397720	3.53959137238561
C	2.82256582306910	0.94197537741068	2.52528347600678
H	2.39493226211822	1.92921737128741	2.56517245147807
C	5.16342228889016	-1.73795055319833	3.01760739776837
H	4.91075016325864	-2.73220348934240	2.65701784211861
H	5.31516719098396	-1.77477122911424	4.09551997607109
H	6.07913505840074	-1.40145811678537	2.53317042163310
C	-6.12666546327027	-0.71739694708499	3.00059358731731
Cl	-6.07033981467884	-2.47742652780094	2.98747398950201
Cl	-6.57393833783845	-0.11729160594645	4.59802636865817
Cl	-7.25419195704927	-0.12015260627372	1.78419188071026
H	-5.13509644361651	-0.34555944644734	2.74755274603574

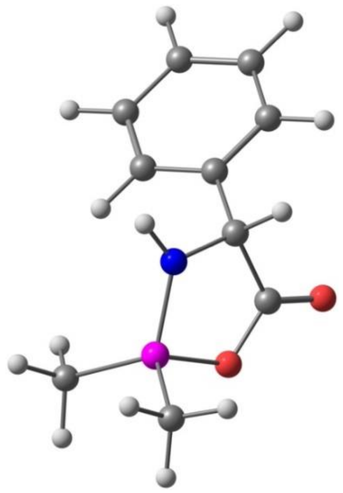

Figure S21.
Optimized molecular structure of **(Phg)SiMe$_2$**.

PBE0:
final single point energy: -884.625592931935 a.u.
final Gibbs free energy: -884.45129273 a.u.

Table S8. Atomic coordinates for optimized structure of **(Phg)SiMe$_2$**.

Si	-0.56850420369631	0.06810583773782	-0.18136754831563
O	-2.24451117740762	0.07644160947686	0.12680069482636
O	-3.69971388432200	0.11400268968165	1.81096421210122
N	-0.18726113822819	0.01271471960995	1.48589340587954
H	0.71067227521875	-0.07232077922077	1.92721629334110
C	-2.56730128290013	0.05291620149795	1.42204237017107
C	-1.34247898025238	-0.05552252048760	2.34247049316374
H	-1.41153100944736	0.80731019839681	3.01665057700977
C	-1.45074916009789	-1.30867247298132	3.18686767803462
C	-2.11522110542741	-1.26131963903548	4.40719376808755
H	-2.52544044088140	-0.31967793850067	4.75456024922453
C	-2.25534677663182	-2.40595567181646	5.17713317094844
H	-2.77336712687086	-2.35534242823907	6.12750840473802
C	-1.72850009062539	-3.61151031902179	4.73424071577396
H	-1.83388699646518	-4.50585695984932	5.33660043752737
C	-1.06049329361349	-3.66320076947857	3.51896149767483
H	-0.64374727054433	-4.59991971944790	3.16794032755018
C	-0.92237547795882	-2.51704025261536	2.74936945789880
H	-0.39301099987936	-2.55720928560637	1.80518768571862
C	-0.09074436735959	1.62686370894047	-1.05478772666676
H	-0.48594435267834	1.64622113338121	-2.07286673483967
H	0.99857883401249	1.69768954847958	-1.11930281214654
H	-0.45882030854031	2.50128900201644	-0.51568025224245
C	-0.12548780222656	-1.42778592206764	-1.17748400564475
H	-0.51578209421618	-2.33598037307651	-0.71519600236838
H	0.96181471312514	-1.51899299551587	-1.24955448016840
H	-0.52035548208542	-1.35346960225801	-2.19325487727721

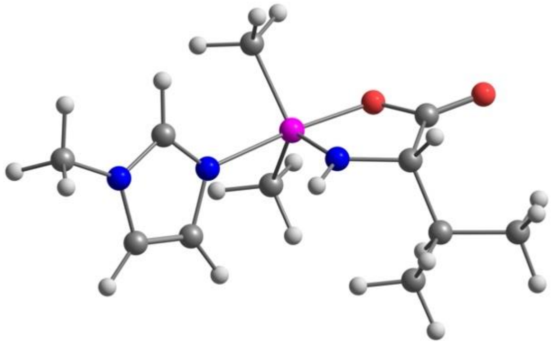

Figure S22.
Optimized molecular structure of **(Val)SiMe$_2$-NMI**.

PBE0:
final single point energy: -1037.076892263604 a.u.
final Gibbs free energy: -1036.80219215 a.u.

Table S9. Atomic coordinates for optimized structure of **(Val)SiMe$_2$-NMI**.

Si	-0.10223496991522	0.03519972255288	0.00203759499821
O	-1.89192533905592	-0.12859903248130	0.25837412852743
O	-3.44980381914456	-0.54596735927377	1.78513788684621
N	0.03678546038735	-0.21671248664894	1.70909806019828
H	0.89985633699227	-0.15027983939442	2.21510852374449
N	1.96924559966106	0.18351362010411	0.04580685360226
N	4.10019319312621	-0.30394899040718	0.00985446999995
C	-2.29658957965627	-0.30769199061642	1.48487162193183
C	-1.16608813637387	-0.17328552830688	2.49727049566405
C	-0.21678001658310	1.73200253767771	-0.79750772085766
H	-1.15886761108363	1.80968869171892	-1.34315362842147
H	0.60331472818324	1.93310492723495	-1.48836884506165
H	-0.21849350300559	2.51799112044589	-0.03676968944946
C	-0.00089809630581	-1.36892365869200	-1.24069858015139
H	0.24944721579895	-2.31215814384542	-0.74657241405997
H	0.73932691403968	-1.18605594567584	-2.02202227073633
H	-0.97336149889208	-1.50062356614492	-1.71663947812834
C	2.85957070232085	-0.74842174705139	-0.23193226897248
H	2.64506602465507	-1.73842651690863	-0.59833482930531
C	3.99888165215937	0.98304473327523	0.47036565948349
H	4.86645581097768	1.56391636541537	0.73351661978005
C	2.66956654286455	1.27434416363115	0.49059524815439
H	2.17389937105141	2.18195050376774	0.79113143349954
C	5.32545822016247	-1.04310402792788	-0.20188232339778
H	5.07472541213307	-2.05871400932355	-0.49894672775287
H	5.90512738710783	-1.07233868433145	0.71968044329593
H	5.91620882013584	-0.57392303411326	-0.98804252631324
H	-1.23634925348116	-1.02654916586000	3.18608749874873
C	-1.31832581622118	1.11603046127290	3.33622948883599
H	-1.43225697593932	1.94753287782358	2.62912709086151
C	-0.06579628888987	1.36636403428857	4.16367454266202
C	-2.53725027139359	1.07146141307256	4.24429172518883
H	-3.45647961479115	0.91833494293803	3.68187139820814
H	-2.44670390165036	0.25351897258914	4.96575555620559
H	-2.62189238809417	2.00309774871125	4.80842477030267
H	0.12263277758093	0.52772236174953	4.84153542760319
H	-0.18851715470909	2.26407218657651	4.77348804893198
H	0.81972506584809	1.50993601115719	3.54222673133318

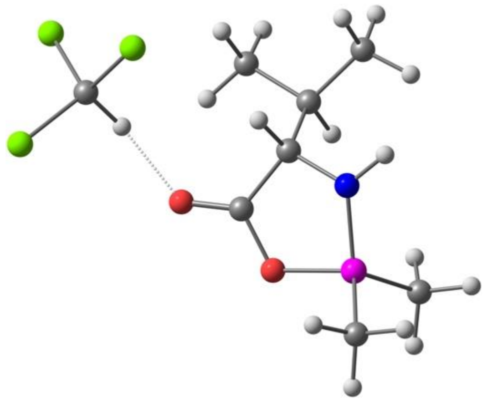

Figure S23.
Optimized molecular structure of **(Val)SiMe$_2$-CHCl$_3$**.

PBE0:
final single point energy: -2197.198991781118 a.u.
final Gibbs free energy: -2197.00676859 a.u.

Table S10. Atomic coordinates for optimized structure of **(Val)SiMe$_2$-CHCl$_3$**.

Si	1.61991810378187	-1.84944534730634	2.85628773314127
O	1.38717926349945	-2.84085943984397	4.22616380960548
O	-0.08462606623560	-3.23268853718643	5.83785974211204
N	0.06419265121399	-1.15190422450547	3.01536925421282
H	-0.40765417740751	-0.56438218091483	2.35195792302689
C	0.18390359943183	-2.71178953159494	4.78460334677870
C	-0.77553083828887	-1.86996095975769	3.95169579855212
C	1.92122344866915	-2.90538564066690	1.36495579001877
H	2.88014229353675	-3.42384186726088	1.43567680785848
H	1.94755241572572	-2.27928894008049	0.46870781490331
H	1.12876906089082	-3.64547854752667	1.24083128282766
C	3.03304226669497	-0.69854203383115	3.16268817339927
H	2.90064421931301	-0.16489903698056	4.10509400852767
H	3.09508995730899	0.03816685898322	2.35698193339605
H	3.98094097832316	-1.24032132261176	3.19193405896218
H	-1.28455010400569	-1.18619987193256	4.64204695854875
C	-1.85328216050287	-2.75808672744895	3.28764042082733
H	-1.32831260888882	-3.52908899488489	2.71020202997530
C	-2.69151628880274	-1.92699463234866	2.32722759756803
C	-2.75642694593188	-3.43042691234136	4.30982192615826
H	-2.20452445726325	-4.07346754788119	4.99353567281282
H	-3.28689046700554	-2.68165417785512	4.90478454868337
H	-3.50645669485299	-4.03800724357789	3.79988151038696
H	-3.18740107176961	-1.10939235829037	2.85905845406215
H	-3.46705815233803	-2.54793619576113	1.87503192910779
H	-2.09928606753551	-1.50207619193995	1.51550323867735
C	-2.08186076753583	-1.88526786955525	7.83996940887604
Cl	-2.66043727020031	-0.35022908954799	7.18788897828924
Cl	-1.08589895165699	-1.61582639101642	9.26561989923418
Cl	-3.43160623039329	-2.95465494317624	8.20892748131968
H	-1.46358193777437	-2.36363910135703	7.08360646814979

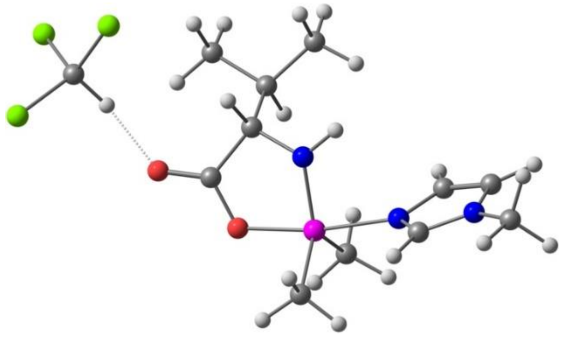

Figure S24.
Optimized molecular structure of **(Val)SiMe$_2$-NMI-CHCl$_3$**.

PBE0:
final single point energy: -2462.754741345294 a.u.
final Gibbs free energy: -2462.46863901 a.u.

Table S11. Atomic coordinates for optimized structure of **(Val)SiMe$_2$-NMI-CHCl$_3$**.

Si	1.55223017468131	-1.56210397572348	2.53239077271352
O	1.37845566515527	-2.55204550731245	4.06164846537453
O	0.02586496948323	-2.97615905721553	5.76621350634563
N	-0.04161303697526	-0.98301642162915	2.89287594889111
H	-0.57339742966016	-0.40980237620702	2.26481429235716
N	1.43058908327158	-0.33369707209691	0.87937444791260
N	1.51085264509803	1.40937236080673	-0.43656939651097
C	0.23858272099204	-2.47246935116752	4.67511593949558
C	-0.80296789801983	-1.69603756955331	3.88447279003369
C	1.89380017396590	-3.05371070580820	1.44247745650437
H	2.46042965162728	-3.79121928838330	2.01351989566346
H	2.45516864544796	-2.80655091757295	0.54043152657875
H	0.95737402541353	-3.53095237868484	1.13986638413976
C	3.10829702824474	-0.68948859770397	3.11378477298944
H	2.86926558238408	0.26243689915509	3.59608348731544
H	3.80437883552191	-0.49202890061556	2.29636784766132
H	3.61667978686985	-1.30965047140124	3.85261428515539
C	1.89656927631036	0.89200081816295	0.73706719359381
H	2.50173887549548	1.42644932724488	1.44999272309278
C	0.75417095060688	0.46529219402798	-1.08047186113031
H	0.32782545883026	0.64312181609535	-2.05294393909052
C	0.70871538059539	-0.61313348935124	-0.25208935756736
H	0.21295970205629	-1.55916155398490	-0.38901754263474
C	1.83761943626675	2.72732534662731	-0.93596064791313
H	2.43333718142615	3.24707095556297	-0.18935663251972
H	0.92469410990339	3.29131005218099	-1.12274135410502
H	2.40963142169435	2.64613365551661	-1.85938391641048
H	-1.31290623783997	-1.01667294896879	4.58058933022528
C	-1.86790673105706	-2.64397451635296	3.28455134471531
H	-1.32746765890748	-3.40773308911390	2.71130796249959
C	-2.77293030622801	-1.88327240242912	2.32612421837307
C	-2.70676810355126	-3.32911666150361	4.35143290071633
H	-2.09868692669328	-3.91556510671753	5.03818111060945
H	-3.25888905189667	-2.58943749760648	4.93793447871109
H	-3.43834759133296	-3.99321153283709	3.88612498249266
H	-3.28665638105587	-1.06880600045947	2.84642347971238
H	-3.53595533532817	-2.54916453378312	1.91745140784455
H	-2.22234383179101	-1.45985007436276	1.48446702516516
C	-2.05682626736598	-2.14639530669511	7.85946173367728
Cl	-2.97382736106903	-0.72250016453200	7.35962499111885
Cl	-1.06466163730481	-1.77768456355662	9.26749883635089
Cl	-3.13989854726029	-3.49523845024302	8.19526845476481
H	-1.39278163533833	-2.43214848256145	7.04243670832000

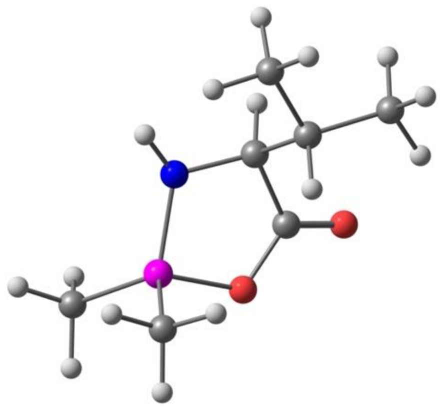

Figure S25.
Optimized molecular structure of **(Val)SiMe$_2$**.

PBE0:
final single point energy: -771.522852221124 a.u.
final Gibbs free energy: -771.34369755 a.u.

Table S12. Atomic coordinates for optimized structure of **(Val)SiMe$_2$**.

Si	-0.53642672853584	-0.01281321478315	-0.03065145943545
O	-2.18638240803342	-0.23678352949508	0.32274757890482
O	-3.53378204294981	-0.71795807045460	2.02375703280564
N	-0.07814051363229	-0.28922979110396	1.59753040931710
H	0.82703602674404	-0.13381648866357	2.00356092919425
C	-2.43904352306515	-0.42661852444365	1.62473991267720
C	-1.20807737810951	-0.21687291776052	2.50212131331037
C	-0.28436541523495	1.69802599508070	-0.69803018926856
H	-0.76471258178952	1.81671322532352	-1.67213642136983
H	0.78293836851822	1.89721240157179	-0.82694579020207
H	-0.69124996015856	2.44435645892142	-0.01327595656569
C	0.03751322167320	-1.29360809067603	-1.23425946679320
H	-0.21390641021434	-2.29669710191797	-0.88653490048757
H	1.12245333749934	-1.23350053094904	-1.35584216508910
H	-0.41568889260515	-1.13528017133605	-2.21546246750858
H	-1.19319487557612	-1.03697705592263	3.23040618339620
C	-1.30946311105657	1.11170991643805	3.28509999430379
H	-1.47024660122056	1.91026381852672	2.55008406395128
C	-0.00345184154987	1.39049337056788	4.01464266328365
C	-2.46609959335106	1.11112350771793	4.27214549323635
H	-3.42699026679987	0.96753915160364	3.78083033088505
H	-2.34300945882345	0.31084286158059	5.00776621217511
H	-2.49385748145172	2.05882664459709	4.81353462380980
H	0.22967627209499	0.57941497581591	4.71115334451791
H	-0.08796579733192	2.31168361677607	4.59414460874905
H	0.83891165495993	1.51116054298490	3.33175212220244

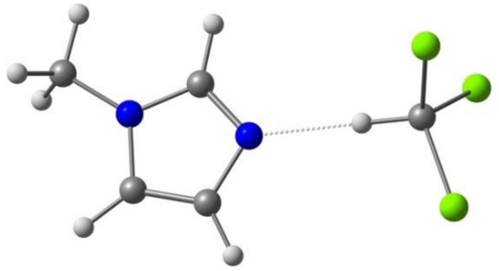

Figure S26.
Optimized molecular structure of **NMI-$CHCl_3$**.

PBE0:
final single point energy: -1691.212264473370 a.u.
final Gibbs free energy: -1691.13013036 a.u.

Table S13. Atomic coordinates for optimized structure of **NMI-$CHCl_3$**.

C	-0.76048763692940	-0.22226184934574	-0.18843621831088
H	0.05063002610744	-0.13836491738186	0.54171500064033
Cl	-1.71498014086543	-1.64852721161745	0.21697887395535
Cl	-1.74434506681817	1.23769192096119	-0.08410663794775
Cl	-0.05149553454934	-0.38243523058371	-1.79490274206662
N	1.47999715282168	0.01680983366774	1.96720290799562
N	3.17410486582311	-0.39591842760884	3.31633571724422
C	2.41512463154107	-0.84580757379618	2.29563481023786
H	2.58715412734326	-1.80507584174499	1.83122031780117
C	2.69072778959197	0.83664241189277	3.66320063538998
H	3.12778562375227	1.41477103286921	4.46021184951234
C	1.64345903718324	1.07590037952903	2.81911693801826
H	1.00358323157171	1.94320028103529	2.77684564859869
C	4.28280087032923	-1.08695339346054	3.93130549049222
H	4.43328916600724	-2.03441339150522	3.41803813299897
H	4.07339861530354	-1.28258576468545	4.98301046414388
H	5.19236456278654	-0.49189349122525	3.85102513629630

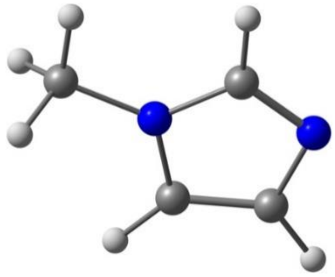

Figure S27.
Optimized molecular structure of **NMI**.

PBE0:
final single point energy: -265.536400689952 a.u.
final Gibbs free energy: -265.46485758 a.u.

Table S14. Atomic coordinates for optimized structure of **NMI**.

N	1.99337240176287	0.05429921370311	0.20549197659312
N	4.16317804897557	-0.20500266289349	-0.12677783754544
C	2.92745568831647	-0.73083123877837	-0.27944574168642
H	2.77229495744862	-1.68941473297887	-0.75225361721750
C	4.00715414175282	1.00091721961695	0.49994335102419
H	4.84801199060323	1.62954534695676	0.74211912462789
C	2.66184173996216	1.14348361710051	0.69668773673521
H	2.14297886918333	1.96709774499015	1.16221859378846
C	5.40911938125823	-0.80505751646774	-0.53890561763150
H	5.19772307672831	-1.76100299202518	-1.01381704013969
H	6.05520739330288	-0.97367860180865	0.32297629703655
H	5.92522737370547	-0.16235973341518	-1.25235308358487

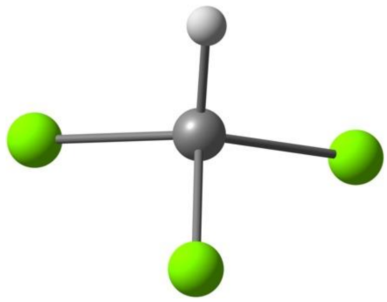

Figure S28.
Optimized molecular structure of **$CHCl_3$**.

PBE0:
final single point energy: -1425.667678253672 a.u.
final Gibbs free energy: -1425.67637414 a.u.

Table S15. Atomic coordinates for optimized structure of **$CHCl_3$**.

C	0.00096893517865	-0.00044281725380	-0.02218018811680
H	0.00001922524628	-0.00012497288538	1.06154555594259
Cl	-0.83736828272343	-1.45047027936703	-0.56337115722042
Cl	-0.83851514347521	1.44790245433247	-0.56503956635292
Cl	1.67695860577371	-0.00019479082625	-0.56318307425245

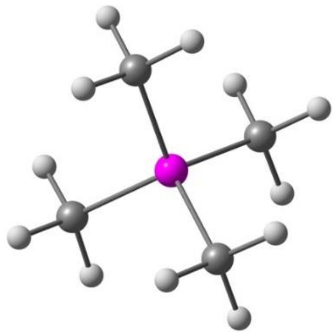

Figure S29.
Optimized molecular structure of **$SiMe_4$**.

PBE0:
final single point energy: -450.025991074705 a.u.
final Gibbs free energy: -449.91135276 a.u.

Table S16. Atomic coordinates for optimized structure of **$SiMe_4$**.

Si	0.00714122583757	-0.00933554648435	-0.02081052932285
C	-1.53590169769129	-0.70678140586966	0.78434486367904
H	-1.56301774895502	-1.79586116543702	0.69625240673790
H	-1.57154008129356	-0.45476507563685	1.84718029693305
H	-2.43875994108642	-0.30926102404289	0.31405358790119
C	1.52218676061079	-0.72779092398927	0.81843325603365
H	1.53474816786991	-0.47833075749753	1.88241291151602
H	1.53803117553011	-1.81693461977911	0.72857012122681
H	2.44104718449280	-0.34035994509016	0.37134817567130
C	0.01825927262681	1.85751093410415	0.15428248157196
H	0.00792913875686	2.15300516206046	1.20640344213145
H	0.91047101686339	2.28804975990319	-0.30729980684061
H	-0.85646017548843	2.30083297345235	-0.32820551491108
C	0.02256641668785	-0.46380437419999	-1.84006277985621
H	0.91447674933791	-0.07078025001423	-2.33449778778393
H	0.01442250158206	-1.54842385804725	-1.97408629025857
H	-0.85252018368134	-0.05548220043182	-2.35195143542913
